# Older adults preserve audiovisual integration through enhanced cortical activations, not by recruiting new regions

**DOI:** 10.1371/journal.pbio.3002494

**Published:** 2024-02-06

**Authors:** Samuel A. Jones, Uta Noppeney

**Affiliations:** 1 Computational Neuroscience and Cognitive Robotics Centre, University of Birmingham, Birmingham, United Kingdom; 2 Department of Psychology, Nottingham Trent University, Nottingham, United Kingdom; 3 Donders Institute for Brain, Cognition & Behaviour, Radboud University, Nijmegen, the Netherlands; Columbia University, UNITED STATES

## Abstract

Effective interactions with the environment rely on the integration of multisensory signals: Our brains must efficiently combine signals that share a common source, and segregate those that do not. Healthy ageing can change or impair this process. This functional magnetic resonance imaging study assessed the neural mechanisms underlying age differences in the integration of auditory and visual spatial cues. Participants were presented with synchronous audiovisual signals at various degrees of spatial disparity and indicated their perceived sound location. Behaviourally, older adults were able to maintain localisation accuracy. At the neural level, they integrated auditory and visual cues into spatial representations along dorsal auditory and visual processing pathways similarly to their younger counterparts but showed greater activations in a widespread system of frontal, temporal, and parietal areas. According to multivariate Bayesian decoding, these areas encoded critical stimulus information beyond that which was encoded in the brain areas commonly activated by both groups. Surprisingly, however, the boost in information provided by these areas with age-related activation increases was comparable across the 2 age groups. This dissociation—between comparable information encoded in brain activation patterns across the 2 age groups, but age-related increases in regional blood-oxygen-level-dependent responses—contradicts the widespread notion that older adults recruit new regions as a compensatory mechanism to encode task-relevant information. Instead, our findings suggest that activation increases in older adults reflect nonspecific or modulatory mechanisms related to less efficient or slower processing, or greater demands on attentional resources.

## Abstract 2

Multisensory perception changes as we get older. This study uses multivariate fMRI to explore the neural mechanisms underlying these changes, revealing that older adults recruit similar brain regions, but increase activations in some of these reasons to preserve their performance.

## Introduction

The effective integration of multisensory signals is central to our ability to successfully interact with the world. Locating and swatting a mosquito, for example, relies on spatial information from hearing, vision, and touch. When signals from different senses are known to come from a common cause, humans typically perform this integration process in a statistically near-optimal way, weighting the contribution of each input by its relative reliability [1–5] (i.e., inverse of variance; though also see, for instance, [6,7]). However, determining specifically which signals share a common cause, and should thus be integrated, is computationally challenging. Young, healthy adults balance sensory integration and segregation in line with the predictions of normative Bayesian causal inference [8–12]: They bind inputs that are close together in space and time but process them independently when they are spatially or temporally disparate and hence unlikely to share a common source. Recent functional magnetic resonance imaging (fMRI) and electroencephalography research has revealed that, for audiovisual spatial signals, these operations take place dynamically across the cortical hierarchy that encompasses primary sensory areas as well as higher-level regions such as intraparietal sulcus and planum temporale [10,13]. Evidence also suggests that they interact with top-down attentional processes [5,14–19].

Normal healthy ageing leads to a variety of sensory and cognitive changes, including loss of sensory acuity [20–22], reduced processing speed [23], and impaired attentional and working memory processes [24,25]. In multisensory perception, ageing has been associated with altered susceptibility to the sound-induced flash and McGurk illusions [26–30]; these age differences may be caused by various computational or neural mechanisms, including changes in sensory acuity, prior binding tendency, and attentional resources (for further discussion, see [31]). By contrast, older adults perform in a way that is comparable to their younger counterparts on audiovisual integration of spatial signals (as indexed by the spatial ventriloquist illusion) [32,33]. They weight and combine sensory signals in ways that are consistent with normative Bayesian causal inference. However, they sacrifice response speed to maintain this audiovisual localisation accuracy [32].

This raises the question of *how* older adults preserve audiovisual integration and spatial localisation accuracy in these intersensory selective attention paradigms. There are 3 possibilities:

First, older adults may engage the same neural mechanisms, in the same way as their younger counterparts, to form neural spatial representations that are similar between age groups. In short, older adults’ preserved behavioural performance is mirrored by preserved neural processing.

Second, older adults may show neural encoding deficits in the key regions engaged by younger adults. To compensate for such deficits, they recruit additional regions. Critically, if such activations are truly compensatory, we would expect age differences not only in the magnitude of the regional blood-oxygen-level-dependent (BOLD) responses but also in their information content: The additional brain activations would encode more task- or stimulus-relevant information in older than in younger participants. We might also expect representations of the stimuli in areas along the dorsal visual and auditory spatial processing hierarchies to be degraded, necessitating such compensatory activity. This compensatory recruitment of extra regions to sustain task performance in older adults has been widely held, in the healthy ageing research field, to explain the additional activations typically found in older adults (see, for example, [34–36]).

Third, older adults may show increased activations that are not directly attributable to compensatory activity. Indeed, the notion of age-related compensatory recruitment has recently been challenged by research into the impact of healthy ageing on memory [37] and motor performance [38]. These studies also observed that older adults activate additional cortical regions while performing tasks. Crucially, however, sophisticated model-based multivariate Bayesian decoding analyses found that these regions did not encode additional information relevant for task performance. The authors therefore concluded that the age-related activation increases may instead reflect nonspecific mechanisms such as reduced neural processing efficiency. In our spatial localisation task, this could mean that older observers suffer from noisier neural coding despite their behavioural performance being largely preserved. For instance, it is increasingly understood that ageing affects auditory temporal processing, with potential associated effects on spatial processing (for instance, interaural time difference cues [39]). As a consequence, and as recently suggested by computational modelling of behavioural data [32], older adults may accumulate noisier sensory information for longer until they reach a decision threshold and commit to a response. This would result in larger BOLD responses in the associated regions [40]. Older adults may additionally, or alternatively, need to exert more top-down attentional control to attenuate internal sensory noise, or engage more cognitive control to inhibit conflicting or irrelevant visual and auditory signals [41]. Common to all these potential mechanisms is that any age-related activation increases would not encode additional stimulus- or task-relevant information in older, compared to younger, adults. Instead, activation increases would reflect more general mechanisms that may help to enhance existing neural encoding in older adults, thereby allowing them to maintain precision and accuracy of spatial representations at the neural and behavioural levels.

To adjudicate between these 3 possibilities, we presented healthy younger and older participants with synchronous audiovisual signals at varying degrees of spatial disparity in a spatial ventriloquist paradigm. In an auditory selective attention task, participants reported the location of the auditory signal, while ignoring the task-irrelevant visual signals (which were spatially congruent or incongruent). First, we investigated whether older and younger observers weight and combine audiovisual signals similarly into spatial representations at the behavioural level. Second, we used multivariate pattern analysis to assess whether observers’ neural spatial representations, decoded from activity patterns along the dorsal visual and auditory spatial processing hierarchies [10,13], were comparable between younger and older adults. Third, we applied whole-brain univariate analyses to identify the neural systems supporting spatial localisation performance more broadly and assessed differences in activation levels between older and younger participants. Finally, using multivariate Bayesian decoding [37,38,42], we assessed whether regions with greater activation in older adults encoded the same amount of stimulus- or task-relevant information (such as visual and auditory location, or their spatial relationship) in both age groups.

## Results

### Audiovisual integration behaviour

**Fig 1:**
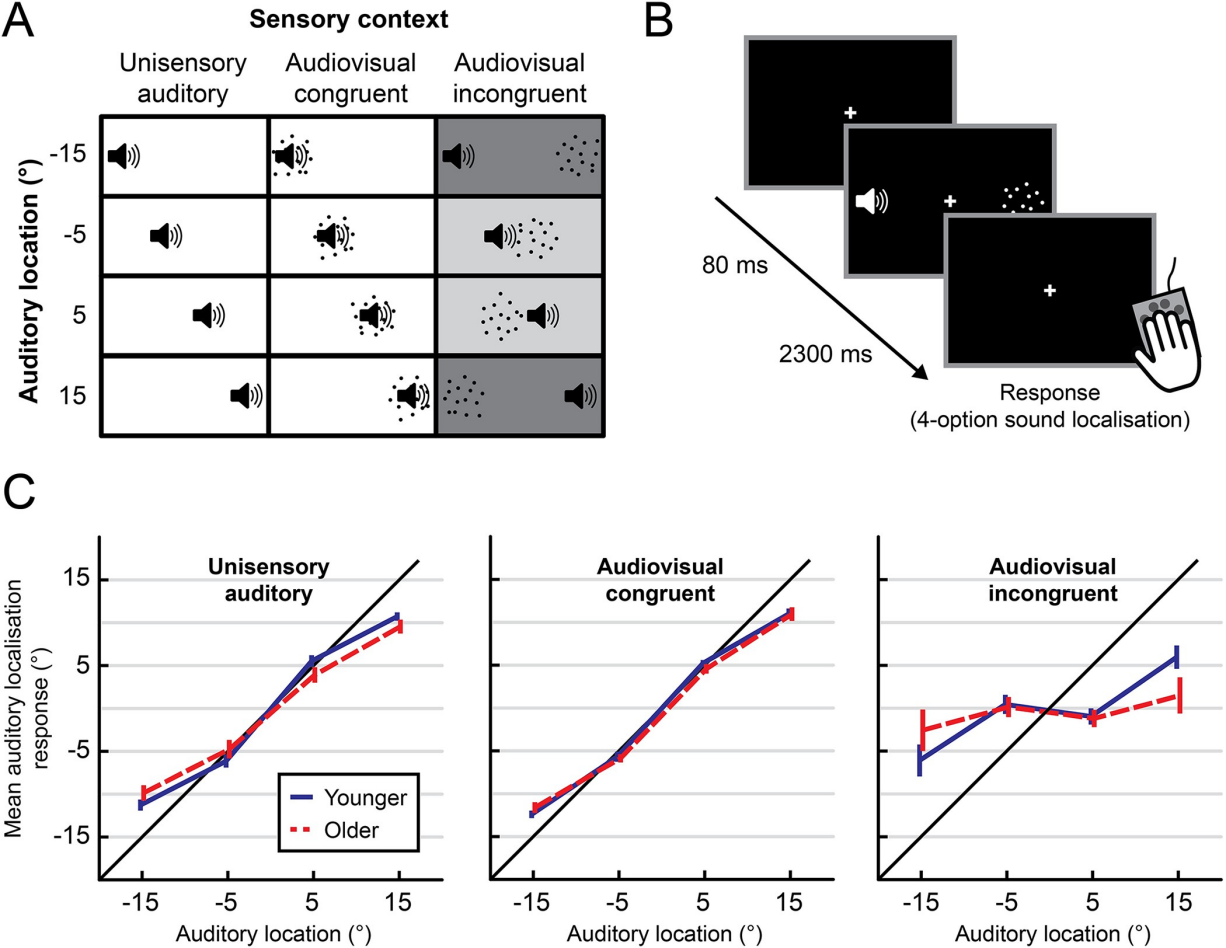
Experimental design and behavioural results. (**A** and **B**) The experiment conformed to a 4 (auditory location) × 3 (sensory context: unisensory auditory, audiovisual congruent, audiovisual incongruent) factorial design. Auditory (white noise bursts) and visual signals (cloud of dots) were sampled from 4 possible azimuthal locations (−15°, −5°, 5°, or 15°). Auditory and visual stimuli were presented either at the same (congruent) or opposite (incongruent) spatial locations, or the auditory stimulus was presented alone (unisensory). Participants reported their perceived location of the sound. (**C**) Across-participants mean (± SEM) perceived sound locations as a function of the true sound location (*x* axis). The data underlying this Figure can be found in S1 Data.

Inside the scanner, participants were presented with synchronous auditory and visual signals at the same (i.e., congruent) or opposite (i.e., incongruent) locations sampled from 4 possible spatial locations along the azimuth. The experimental design thus conformed to a 4 (auditory location: −15°, −5°, 5°, or 15° azimuth) × 3 (sensory context: unisensory auditory, audiovisual congruent, audiovisual incongruent) factorial design (see Fig 1B). On each trial, participants reported their perceived sound location as accurately as possible by pressing one of 4 spatially corresponding buttons with their right hand. As shown in Fig 1C, both younger and older adults can locate unisensory auditory and audiovisual congruent stimuli quite accurately, though we observe a small central bias for stimuli presented at the most eccentric locations. On audiovisual incongruent trials, their reported sound location is biased by—i.e., shifted towards—the location of the co-occurring visual signal. Crucially, this crossmodal bias is stronger for small audiovisual spatial disparities (5° eccentricity) than for large audiovisual spatial disparities (15° eccentricity). Thus, both younger and older adults combine audiovisual signals in a way that is consistent with the computational principles of Bayesian causal inference: They integrate audiovisual signals when they are close in space and hence likely to come from one source, but segregate those with larger spatial disparities. However, at large spatial disparities, we observe a small trend towards greater crossmodal biases for older than for younger observers.

Consistent with these impressions, a 2 (hemifield: left or right) × 2 (eccentricity: 5° or 15°) × 3 (sensory context: unisensory auditory, audiovisual congruent, or audiovisual incongruent) × 2 (age group: younger or older) mixed ANOVA on localisation responses identified significant main effects of eccentricity and sensory context (see Table 1). Moreover, a small three-way (eccentricity × sensory context × age) interaction was observed. This likely reflects a stronger visual influence on perceived sound location in older adults for audiovisual stimuli at large spatial disparities (see right panel of Fig 1C), suggesting older observers’ ability to segregate audiovisual signals is slightly inferior to that of younger adults. Potentially, this small difference across age groups may result from subtle age-related decreases in auditory spatial reliability, which become apparent in challenging sound localisation tasks with interfering spatially disparate visual signals. However, no follow-up *t* tests that separately compared the age groups in each condition reached statistical significance, *p* > .05 (see Table A in S1 Text for full results, including Bayes factors). No other significant effects were observed.

**Table 1:** Results of mixed ANOVA on mean auditory localisation responses during the spatial ventriloquist task.

	*df*					
	effect	error	*F*	*p*	*η* ^2^_*p*_	*BF*_*excl*_
Hemifield	1	30	1.301	.263	.042	8.401
Hemifield × Age	1	30	0.038	.848	.001	131.142
**Eccentricity**	**1**	**30**	**263.039**	**< .001**	**.898**	**<0.001**
Eccentricity × Age	1	30	3.971	.055	.117	0.605
**Sensory context**	**1.145**	**34.346**	**51.106**	**< .001**	**.630**	**<0.001**
Sensory context × Age	1.145	34.346	0.651	.445	.021	0.936
Hemifield × Eccentricity	1	30	0.087	.770	.003	9.117
Hemifield × Eccentricity × Age	1	30	0.624	.436	.020	>1,000
Hemifield × Sensory context	1.944	58.333	0.176	.833	.006	14.106
Hemifield × Sensory context × Age	1.944	58.333	0.265	.762	.009	573.891
Eccentricity × Sensory context	1.203	36.088	2.037	.160	.064	0.711
**Eccentricity** × **Sensory context** × **Age**	**1.203**	**36.088**	**5.330**	**.021**	**.151**	**0.270**
Hemifield × Eccentricity × Sensory context	1.471	44.126	0.278	.690	.009	258.527
Hemifield × Eccentricity × Sensory context × Age	1.471	44.126	1.219	.294	.039	>1,000
Age	1	30	2.196	.149	.068	1.452

*Greenhouse–Geisser correction applied to all within-participants tests with* df_*effect*_ *> 1*. BF_*excl*_ *is based on an equivalent Bayesian ANOVA; greater values indicate more evidence that a given term does* not *have predictive value within the model (see Materials and methods for more details).*

Overall, these behavioural results suggest that older and younger adults combine auditory and visual signals into spatial representations in a way that is consistent with Bayesian causal inference. They also suggest that the age groups are largely comparable in their visual and auditory spatial precision.

### fMRI results

#### Decoding spatial representations from fMRI activation patterns along audiovisual pathways

Next, we used fMRI decoding methods to investigate whether older and younger adults integrate auditory and visual signals into comparable spatial representations at the neural level, thereby mirroring the behavioural pattern. More specifically, we asked whether older adults assign similar weights to auditory and visual signals when combining them into neural representations along the auditory and visual spatial processing hierarchies that have been identified in previous research on younger adults [5,10,13,14,43]. To address this question, we trained support vector regression models to learn the mapping between regional fMRI activation patterns and external spatial locations, specifically for audiovisual *congruent* trials. We then applied those trained support vector regression models to the activation patterns evoked by audiovisual *incongruent* trials (as well as to unisensory auditory and to different audiovisual congruent trials).

This was performed separately in multiple regions across the auditory and visual spatial processing hierarchies. In visually dominant regions, the decoded spatial locations for audiovisual incongruent trials should largely reflect the true location of the visual stimulus. Similarly, in auditory dominant regions, the decoded spatial locations for audiovisual incongruent trials should reflect the true location of the auditory stimulus. Crucially, in regions with crossmodal influences, the decoded locations should be influenced by both auditory and visual locations. This analysis approach thus allows us to investigate how specific brain regions weigh and integrate auditory and visual signals, rather than just addressing the final reported location via behavioural responses.

**Fig 2:**
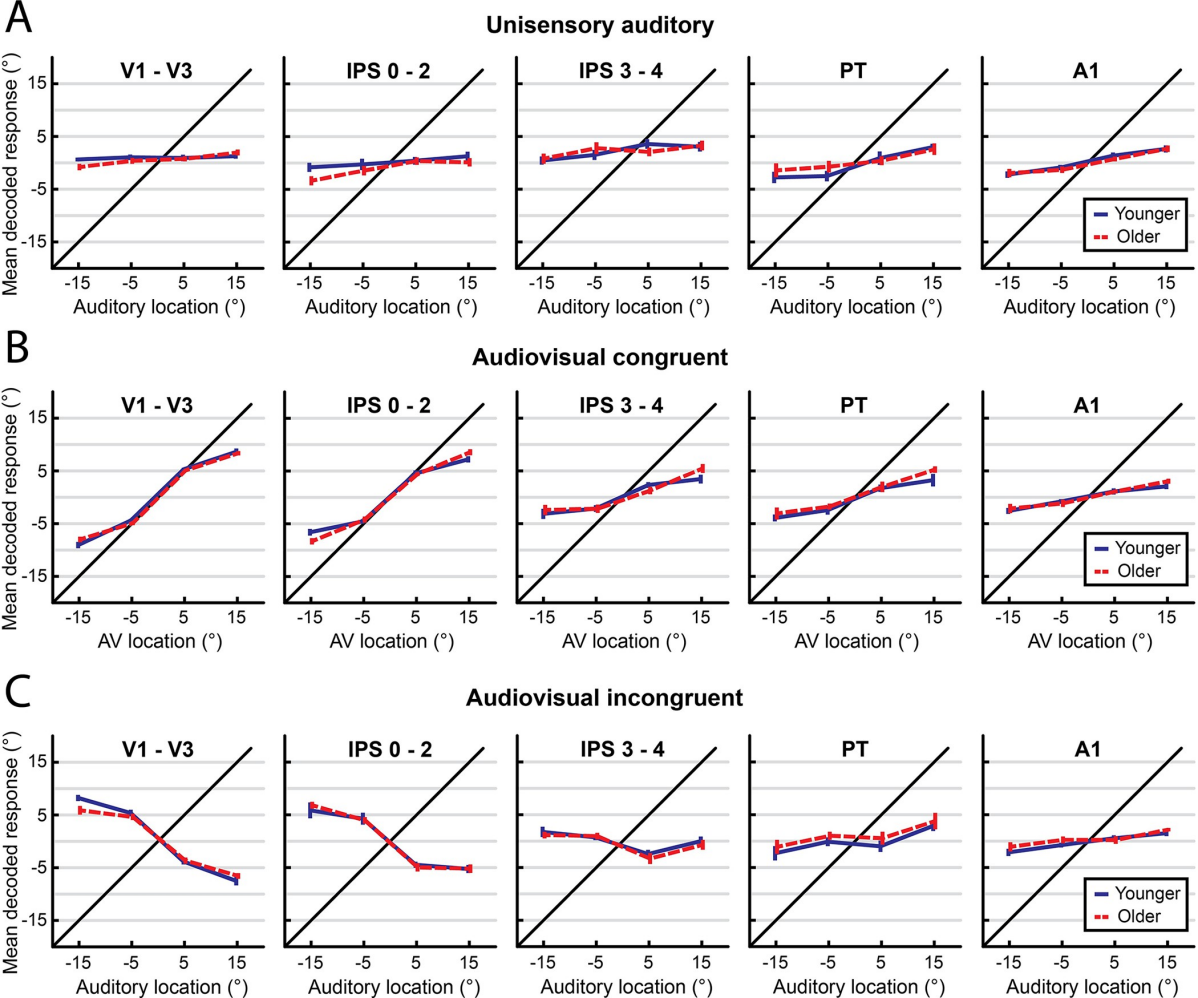
fMRI multivariate decoding results (support vector regression). Across-participants mean (±1 SEM) decoded spatial locations for younger (blue) and older (red) participants for (**A**) unisensory auditory, (**B**) congruent audiovisual, and (**C**) incongruent audiovisual stimuli. Results for 5 ROIs are shown: visual regions (V1-V3); posterior intraparietal sulcus (IPS 0–2); anterior intraparietal sulcus (IPS 3–4); planum temporale (PT); and primary auditory cortex (A1). Note that for incongruent conditions, results for all ROIs are plotted according to the location of the auditory stimulus. The data underlying this Figure can be found in S1 Data.

Fig 2 shows the spatial locations decoded with support vector regression from regional BOLD response patterns for unisensory auditory, congruent audiovisual, and incongruent audiovisual stimuli along the dorsal auditory and visual spatial processing hierarchies identified in previous research [5,10,13,14,43]. As previously reported for younger populations [10,13], primary auditory area A1 and “higher-level” auditory area planum temporale encoded mainly the sound location, while “low-level” visual areas V1-V3, posterior intraparietal sulcus, and anterior intraparietal sulcus represented the visual location. As anticipated, decoding accuracy for visual stimulus location (which is encoded retinotopically [44]) was far higher than for auditory stimulus location (which is encoded across broadly tuned neural populations [45]). Further, the decoding accuracy for audiovisual congruent stimuli was smaller for parietal than occipital visual areas, reflecting the increase in receptive field sizes along the visual processing hierarchy.

Most importantly, the comparison between unisensory auditory, congruent audiovisual, and incongruent audiovisual conditions provides insights into how different regions combine auditory and visual signals.

In planum temporale, congruent visual inputs increased decoding accuracy compared to unisensory auditory conditions. Conversely, incongruent visual inputs biased auditory spatial encoding mainly at small spatial disparities (i.e., a “neural ventriloquist effect”). These crossmodal biases broke down at large spatial disparities, when the brain infers that 2 signals come from different sources, thereby mirroring the integration profile observed at the behavioural level.

In visual areas, we observed an influence of a displaced sound on the decoded spatial location mainly at large spatial disparities. This pattern may be explained by the fact that, at small spatial disparities, observers experience a ventriloquist illusion and thus perceive the sound shifted towards the visual signal. By contrast, at large spatial disparities (when observers are less likely to experience a ventriloquist illusion), a displaced sound from the opposite hemifield biases the spatial encoding in visual cortices via mechanisms of top-down attention. As previously reported [5,10,13,14,43], these crossmodal interactions increased across the cortical hierarchy, being more pronounced in intraparietal sulcus and planum temporale than in early visual and auditory cortices.

These impressions were confirmed statistically by applying the same analyses used to assess behavioural responses: 2 (hemifield: left or right) × 2 (eccentricity: 5° or 15°) × 3 (sensory context: unisensory auditory, audiovisual congruent, or audiovisual incongruent) × 2 (age group: younger or older) mixed ANOVAs were conducted on decoded spatial estimates, separately for each region of interest (ROI) along the visual and auditory processing hierarchy (Table 2). Here, we report results after Bonferroni correction for multiple comparisons in 5 regions; see Table E in S1 Text for uncorrected values. We observed main effects of, and/or interactions with, stimulus eccentricity in all ROIs, confirming that all regions encoded information about the location of the stimuli. Importantly, significant effects of sensory context were apparent in all ROIs except primary auditory cortex, suggesting that all regions except A1 held at least some information about whether a visual stimulus was present or its spatial congruence with the sound. We confirmed that these sensory context effects were not driven entirely by differences between unisensory auditory versus audiovisual stimuli: follow-up ANOVAs that excluded the unisensory condition, so 2 (hemifield: left or right) × 2 (eccentricity: 5° or 15°) × 2 (congruence: audiovisual congruent or audiovisual incongruent) × 2 (age group: younger or older), still showed a significant main effect of congruence and/or an eccentricity × congruence interaction in all ROIs except A1 (for detailed results, see Tables F-J in S1 Text).

**Table 2:** Results of ANOVAs on support vector regression decoded responses in 5 ROIs.

	*df*					
	effect	error	*F*	*p* (Bonf. corr.)	*η* ^2^_*p*_	*BF*_excl_
*V1-V3*						
Hemifield	1	30	5.820	.110	.162	0.425
Hemifield × Age	1	30	0.744	>.999	.024	2.458
**Eccentricity**	**1**	**30**	**117.363**	**< .001**	**.796**	**<0.001**
Eccentricity × Age	1	30	0.874	>.999	.028	1.912
**Sensory context**	**1.568**	**47.036**	**328.707**	**< .001**	**.916**	**<0.001**
Sensory context × Age	1.568	47.036	5.281	.070	.150	0.355
Hemifield × Eccentricity	1	30	2.448	.640	.075	1.393
Hemifield × Eccentricity × Age	1	30	0.109	>.999	.004	8.696
Hemifield × Sensory context	1.753	52.597	3.500	.215	.104	0.639
Hemifield × Sensory context × Age	1.753	52.597	0.594	>.999	.019	3.786
**Eccentricity** × **Sensory context**	**1.620**	**48.588**	**22.205**	**< .001**	**.425**	**<0.001**
Eccentricity × Sensory context × Age	1.620	48.588	3.165	.305	.095	0.616
Hemifield × Eccentricity × Sensory context	1.666	49.985	1.437	>.999	.046	1.645
Hemifield × Eccentricity × Sensory context × Age	1.666	49.985	1.395	>.999	.044	37.023
Age	1	30	0.386	>.999	.013	1.555
*Posterior intraparietal sulcus (IPS 0–2)*						
Hemifield	1.000	30.000	.039	>.999	.001	35.532
Hemifield × Age	1.000	30.000	.432	>.999	.014	53.582
**Eccentricity**	**1.000**	**30.000**	**47.714**	**< .001**	**.614**	**<0.001**
Eccentricity × Age	1.000	30.000	2.075	.800	.065	6.011
**Sensory context**	**1.656**	**49.671**	**108.823**	**< .001**	**.784**	**<0.001**
Sensory context × Age	1.656	49.671	.170	>.999	.006	13.756
Hemifield × Eccentricity	1.000	30.000	.536	>.999	.018	5.447
Hemifield × Eccentricity × Age	1.000	30.000	.170	>.999	.006	42.344
Hemifield × Sensory context	1.710	51.315	1.234	>.999	.040	5.291
Hemifield × Sensory context × Age	1.710	51.315	1.457	>.999	.046	33.293
**Eccentricity** × **Sensory context**	**1.603**	**48.084**	**9.836**	**.003**	**.247**	**0.008**
Eccentricity × Sensory context × Age	1.603	48.084	1.140	>.999	.037	11.146
Hemifield × Eccentricity × Sensory context	1.934	58.032	4.956	.055	.142	1.925
Hemifield × Eccentricity × Sensory context × Age	1.934	58.032	3.392	.210	.102	>1,000
Age	1	30	1.845	.925	.058	11.370
*Anterior intraparietal sulcus (IPS 3–4)*						
Hemifield	1	30	6.420	.085	.176	<0.001
Hemifield × Age	1	30	< .001	>.999	< .001	11.163
Eccentricity	1	30	5.152	.155	.147	0.006
Eccentricity × Age	1	30	1.894	.895	.059	10.467
**Sensory context**	**1.711**	**51.345**	**14.072**	**< .001**	**.319**	**<0.001**
Sensory context × Age	1.711	51.345	0.954	>.999	.031	18.119
**Hemifield** × **Eccentricity**	**1**	**30**	**7.857**	**.045**	**.208**	**0.012**
Hemifield × Eccentricity × Age	1	30	1.995	0.84	.062	12.914
**Hemifield** × **Sensory context**	**1.758**	**52.737**	**13.737**	**< .001**	**.314**	**<0.001**
Hemifield × Sensory context × Age	1.758	52.737	0.437	>.999	.014	32.517
**Eccentricity** × **Sensory context**	**1.841**	**55.234**	**8.495**	**.004**	**.221**	**0.002**
Eccentricity × Sensory context × Age	1.841	55.234	1.210	>.999	.039	34.829
Hemifield × Eccentricity × Sensory context	1.947	58.422	4.627	.070	.134	0.004
Hemifield × Eccentricity × Sensory context × Age	1.947	58.422	1.228	>.999	.039	92.673
Age	1	30	0.125	>.999	.004	18.601
*Planum temporale (PT)*						
Hemifield	1	30	0.189	>.999	.006	9.322
Hemifield × Age	1	30	1.240	>.999	.040	14.982
**Eccentricity**	**1**	**30**	**31.000**	**< .001**	**.508**	**0.003**
Eccentricity × Age	1	30	0.112	>.999	.004	15.161
**Sensory context**	**1.841**	**55.227**	**10.694**	**< .001**	**.263**	**0.081**
Sensory context × Age	1.841	55.227	1.275	>.999	.041	18.890
Hemifield × Eccentricity	1	30	4.591	.200	.133	3.641
Hemifield × Eccentricity × Age	1	30	0.077	>.999	.003	83.677
Hemifield × Sensory context	1.955	58.650	0.701	>.999	.023	14.915
Hemifield × Sensory context × Age	1.955	58.650	0.238	>.999	.008	547.346
Eccentricity × Sensory context	1.848	55.427	2.129	.660	.066	2.993
Eccentricity × Sensory context × Age	1.848	55.427	0.285	>.999	.009	378.394
Hemifield × Eccentricity × Sensory context	1.971	59.138	0.069	>.999	.002	176.626
Hemifield × Eccentricity × Sensory context × Age	1.971	59.138	0.284	>.999	.009	>1,000
Age	1	30	0.216	>.999	.007	16.997
*A1*						
Hemifield	1	30	0.173	>.999	.006	20.998
Hemifield × Age	1	30	0.334	>.999	.011	37.346
**Eccentricity**	**1**	**30**	**21.772**	**< .001**	**.421**	**0.016**
Eccentricity × Age	1	30	0.092	>.999	.003	18.084
Sensory context	1.857	55.713	4.239	.110	.124	4.259
Sensory context × Age	1.857	55.713	0.646	>.999	.021	41.044
Hemifield × Eccentricity	1	30	0.526	>.999	.017	18.068
Hemifield × Eccentricity × Age	1	30	3.391	.375	.102	179.325
Hemifield × Sensory context	1.858	55.750	0.009	>.999	< .001	72.881
Hemifield × Sensory context × Age	1.858	55.750	1.193	>.999	.038	>1,000
Eccentricity × Sensory context	1.995	59.855	0.044	>.999	.001	21.128
Eccentricity × Sensory context × Age	1.995	59.855	0.155	>.999	.005	>1,000
Hemifield × Eccentricity × Sensory context	1.832	54.949	0.173	>.999	.006	>1,000
Hemifield × Eccentricity × Sensory context × Age	1.832	54.949	0.066	>.999	.002	>1,000
Age	1	30	0.110	>.999	.004	22.037

*Greenhouse–Geisser correction applied to all within-participants tests with* df_*effect*_ *> 1*. BF_*excl*_ *is based on an equivalent Bayesian ANOVA; greater values indicate more evidence that a given term does* not *have predictive value within the model (see Materials and methods for more details).* p *values are Bonferroni corrected for 5 ROIs*.

Some significant effects of hemifield were observed specifically in anterior intraparietal sulcus: both hemifield × eccentricity and hemifield × sensory context interactions were found, indicating a degree of left/right bias in the decoded stimulus locations in this region.

Crucially, however, we observed no significant effect of age on the locations decoded from the activation patterns along the auditory and visual spatial processing hierarchies (see Fig 2 and Table 2). Collectively, these results compellingly demonstrate that younger and older adults combine auditory and visual signals into spatial representations along the auditory and visual processing hierarchies in accordance with similar Bayesian computational principles, further supporting the conclusions from our behavioural analysis.

#### Identification of neural systems involved in spatial localisation of audiovisual signals

**Fig 3:**
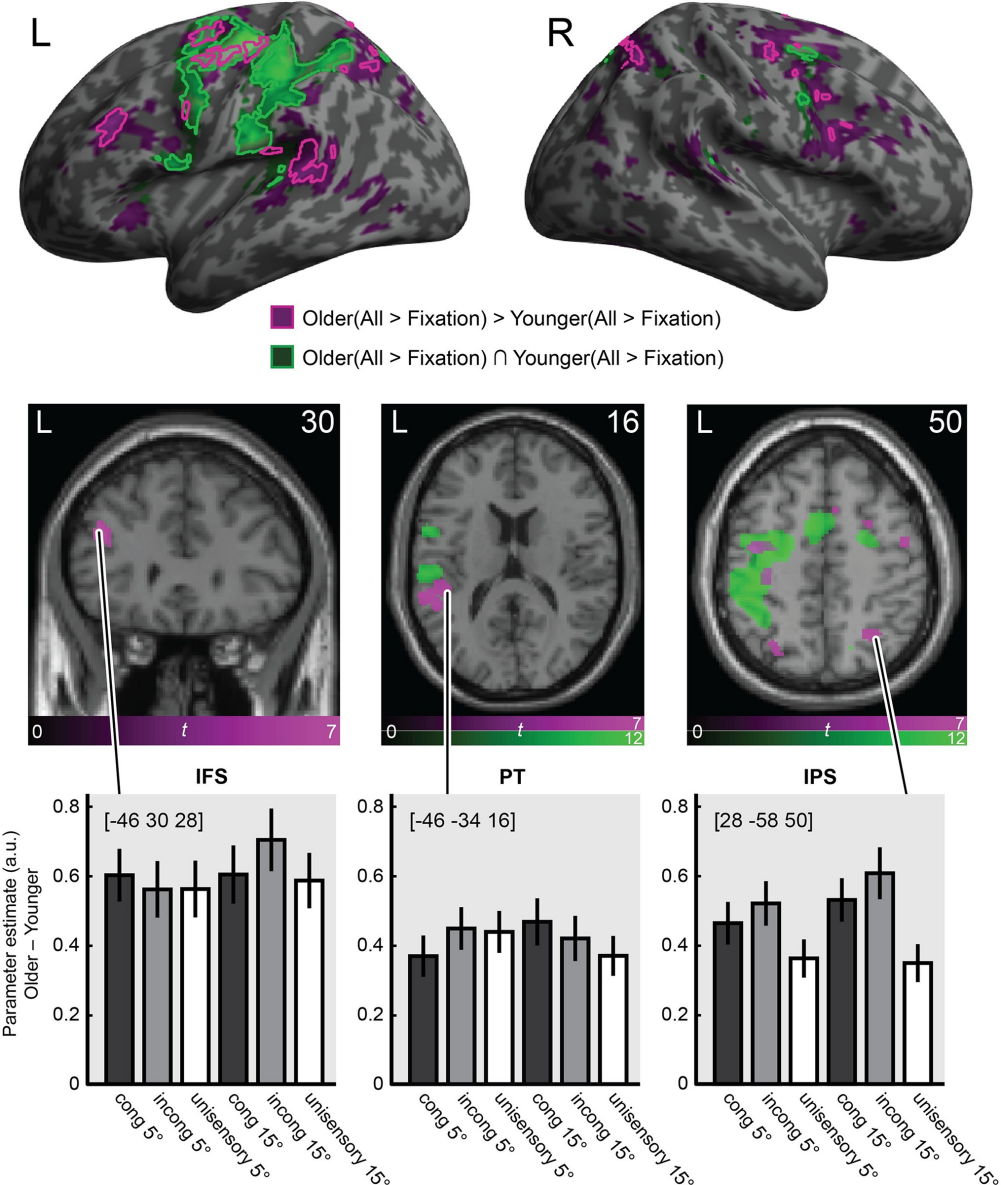
fMRI activation results for older and younger adults. Activations for all stimuli (i.e., pooled over auditory, audiovisual congruent, and incongruent) relative to fixation are rendered on an inflated canonical brain (top row) and coronal/transverse sections (middle row). Green = conjunction over both age groups (All_Older_ > Fixation_Older_) ∩ (All_Younger_ > Fixation_Younger_). Purple = age related activation increases (All_Older_ > Fixation_Older_) > (All_Younger_ > Fixation_Younger_). For inflated brain: bright outlines = height threshold *p* < .05 whole-brain familywise error corrected. For visualisation purposes, we also show activations at *p* < .001, uncorrected, as darker filled areas. Extent threshold k > 0 voxels). For brain sections, height threshold *p* < .05 whole-brain familywise error corrected. Bottom row: Bar plots show mean (±1 SEM) age differences in parameter estimates (arbitrary units) for audiovisual congruent, audiovisual incongruent, and unisensory auditory stimuli at 5° and 15° eccentricities, pooled over left and right stimulus locations, at the indicated peak MNI coordinates. Three illustrative anatomical regions are shown: left inferior frontal sulcus (IFS), left planum temporale (PT), and right intraparietal sulcus (IPS). The data underlying this Figure can be found in S2 Data.

**Fig 4:**
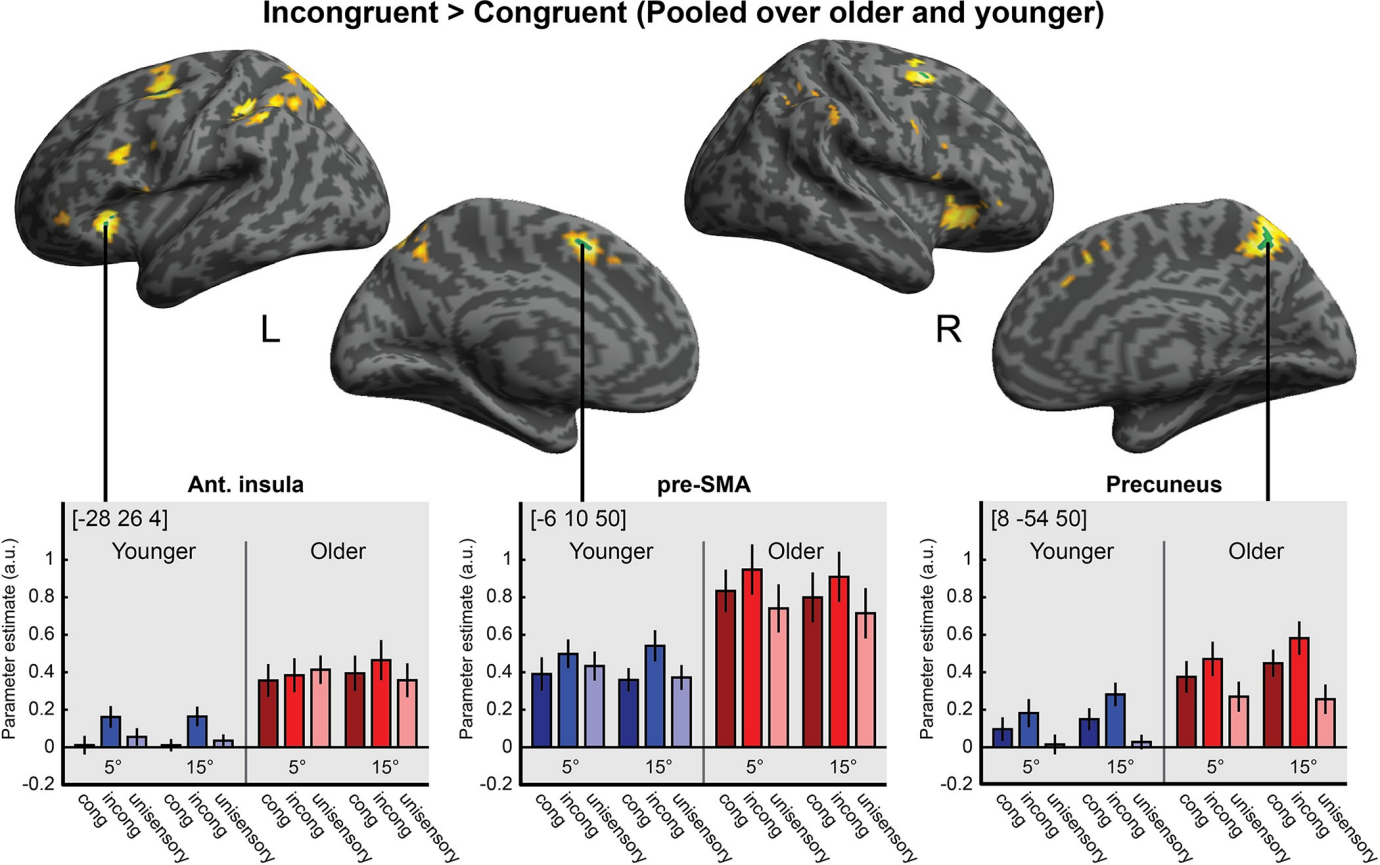
Activation increases for incongruent > congruent audiovisual stimuli. Activation increases for incongruent relative to congruent stimuli (pooled over age groups) are rendered on an inflated canonical brain. Green areas = height threshold *p* < .05, whole-brain familywise error corrected. For visualisation purposes, we also show activations at *p* < .001, uncorrected, in yellow. Bar plots show parameter estimates (across-participants mean ± 1 SEM; arbitrary units) for congruent, incongruent, and unisensory stimuli at 5° and 15° eccentricities, pooled over left and right, at the indicated MNI peak coordinates in 3 anatomical regions: left anterior insula, left pre-supplementary motor area (pre-SMA), and right precuneus. The data underlying this Figure can be found in S2 Data.

The behavioural and neuroimaging analyses reported so far provide convergent evidence that older and younger adults combine audiovisual signals into spatial representations in a similar way. These analyses focused selectively on observers’ spatial representations, obtained either directly from their behavioural reports or via neural decoding of BOLD responses along the auditory and visual spatial processing hierarchies. Next, we asked more broadly which neural systems are engaged in localisation tasks. Do older and younger adults engage overlapping or partly distinct neural systems for audiovisual spatial processing? Do the activation levels differ across age groups in particular regions? To define these task- and stimulus-related processes most broadly, we compared all stimulus conditions to fixation (i.e., all stimulus conditions > fixation) using mass-univariate general linear model analysis. Moreover, we assessed the neural underpinnings of cognitive control and attentional operations that are critical for localising a sound when presented together with a spatially displaced visual signal (i.e., incongruent > congruent audiovisual stimuli; see Table 3 and Figs 3 and 4, for details).

**Table 3:** Mass univariate fMRI analysis—Results.

Region	Coordinates			*z*-score	*p*-value (FWE*)
*O(All > Fixation) ∩ Y(All > Fixation)*					
R. cerebellum	22	−54	−24	>8	< .001
R. cerebellum	6	−62	−16	6.9	< .001
R. cerebellum	8	−72	−16	5.9	< .001
L. precentral gyrus	−36	−20	64	>8	< .001
L. precentral sulcus	−32	−4	58	>8	< .001
L. intraparietal sulcus	−46	−34	42	>8	< .001
L. supplementary motor area	−4	0	56	>8	< .001
R. superior frontal sulcus	24	−2	50	5.7	< .001
L. thalamus	−14	−18	6	5.4	0.002
L. intraparietal sulcus	−18	−68	54	5.4	0.002
R. precentral gyrus	52	4	42	5.1	0.005
L. planum temporale	−40	−36	10	5.1	0.007
L. anterior insula	−30	18	8	5.0	0.009
L. superior frontal gyrus	−16	−6	68	5.0	0.011
R. intraparietal sulcus	14	−66	52	4.9	0.014
R. superior temporal gyrus	58	−34	14	4.8	0.027
*Incong > Cong (Pooled over age groups)*					
R. precuneus	8	−54	50	5.2	< .001
L. supplementary motor area	−6	10	50	5.0	< .001
L. superior frontal sulcus	−26	6	58	5.0	< .001
L. superior frontal sulcus	−26	−2	48	4.9	< .001
L. anterior insula	−28	26	4	5.0	< .001
R. superior frontal sulcus	24	2	54	4.8	< .001
R. anterior insula	32	26	−4	4.8	< .001
L. superior frontal sulcus	−30	−2	62	4.7	< .001
*O(All > Fixation) > Y(All > Fixation)*					
L. inferior frontal sulcus	−46	30	28	7.3	< .001
L. precentral gyrus	−38	−8	54	6.6	< .001
L. supplementary motor area	−8	−8	64	6.3	< .001
L. superior frontal sulcus	−20	−8	56	5.8	< .001
L. superior temporal gyrus	−60	−40	12	5.8	< .001
L. planum temporale	−46	−34	16	5.6	.001
L. supramarginal gyrus	−50	−44	22	5.4	.001
R. intraparietal sulcus	28	−58	50	5.6	.001
R. precuneus	12	−62	62	5.5	.001
R. intraparietal sulcus	24	−62	56	5.0	.011
R. precentral sulcus	48	−4	52	5.6	.001
R. supplementary motor area	8	18	46	5.5	.001
R. inferior frontal sulcus	36	2	36	5.4	.002
L. precuneus	−10	−64	58	5.3	.002
L. intraparietal sulcus	−26	−70	50	5.2	.004
R. superior frontal sulcus	26	−6	56	5.2	.004
R. supplementary motor area	10	6	56	5.2	.005
R. superior frontal sulcus	26	6	54	5.2	.005
L. precentral sulcus	−46	6	34	5.1	.007
L. precentral sulcus	−50	−8	46	5.0	.012
L. intraparietal sulcus	−28	−54	46	4.9	.014
L. superior temporal pole	−52	14	−4	4.9	.018
R. inferior frontal sulcus	38	14	26	4.9	.019
L. intraparietal sulcus	−24	−62	58	4.8	.031
L. intraparietal sulcus	−44	−40	34	4.7	.037
L. anterior insula	−30	24	0	4.7	.047

*p values whole-brain corrected for familywise errors at the voxel level.

*Effects of stimuli and task relative to fixation*. A conjunction analysis over age groups revealed stimulus-induced activations in a widespread neural system encompassing key areas of the auditory spatial processing hierarchy such as left planum temporale, extending into left inferior parietal lobe and intraparietal sulci bilaterally *(All*_*Older*_ *> Fixation*_*Older*_*) ∩ (All*_*Younger*_ *> Fixation*_*Younger*_*)* [46,47]. At a lower threshold of significance, we also observed stimulus-induced activations in the right hemisphere from right planum temporale into inferior parietal lobe and bilateral insulae. Moreover, we observed common activations related to response selection and motor processing in left precentral gyrus/sulcus and right cerebellum.

Next, we identified regions with greater activations for older relative to younger adults by testing for the interaction *(All*_*Older*_ *> Fixation*_*Older*_*) > (All*_*Younger*_ *> Fixation*_*Younger*_*)*. We observed activation increases for older adults in dorsolateral prefrontal cortices along the inferior frontal sulcus. Interestingly, increased activations for older adults were often found adjacent to the regions that were commonly activated for both groups. For instance, we observed greater activations in the lateral plana temporalia extending into more posterior superior temporal cortices. Likewise, the parietal activations extended from the areas observed for both age groups more posteriorly. Moreover, older adults showed increased activations in the inferior frontal sulcus, a region previously implicated in cognitive control of audiovisual processing tasks [40,48]. In summary, older adults showed increased activations relative to younger adults along the spatial auditory pathways from temporal to parietal and frontal cortices.

The opposite contrast *(All*_*Younger*_ *> Fixation*_*Younger*_*) > (All*_*Older*_ *> Fixation*_*Older*_*)* revealed no activations that were significantly greater in the younger age group.

Overall, these results suggest that older adults sustain spatial localisation performance by increasing activations in a widespread neural system encompassing regions typically associated with auditory spatial processing, such as planum temporale, and in regions associated with attention and executive functions, such as parietal cortices and insulae.

*Effects of audiovisual spatial incongruency*. Consistent with previous research [14,40,48,49], incongruent relative to congruent audiovisual stimuli increased activations in a widespread attentional and cognitive control system including medial and lateral posterior parietal cortices, inferior frontal sulcus and bilateral anterior insulae (i.e., *Incong > Cong*, pooled over age groups). However, none of these incongruence effects significantly interacted with age group after whole-brain correction *(Incong*_*Older*_ *> Cong*_*Older*_*) > (Incong*_*Younger*_ *> Cong*_*Younger*_*)* or *(Incong*_*Younger*_ *> Cong*_*Younger*_*) > (Incong*_*Older*_ *> Cong*_*Older*_*)*.

#### Quantifying stimulus-relevant information in task-related BOLD responses

The activation increases for older relative to younger adults raise the critical question of whether/how they contribute to sound localisation performance in older adults. Do these age-related activation increases encode information about task-relevant variables such as stimulus location or audiovisual congruency, thereby enabling older adults to maintain localisation accuracy? Further, do they encode information that is redundant or complementary to that encoded in brain areas jointly activated by both age groups? To address these questions, we used model-based multivariate Bayesian decoding. This approach treats different sets of brain regions as models to predict target variables (such as stimulus location) and provides an approximation to the log model evidence, which trades off a model’s accuracy in predicting a target variable with its complexity. Therefore, unlike discriminative approaches such as support vector regression, multivariate Bayesian decoding allows one to assess the relative contributions of different regions (and their combinations) to encoding target variables—such as stimulus location or congruence—using standard procedures of Bayesian model comparison.

Specifically, we compared the predictive ability of 3 candidate sets of regions: (i) the regions activated jointly by older and younger adults [O∩Y]; (ii) the regions activated more by older than younger adults [O>Y]; and (iii) the union of the two [O>Y ∪ O∩Y]. To match the number of features across these 3 sets, we limited each set of regions to the most significant 1,000 voxels (see Materials and methods for details).

We computed multivariate Bayesian decoding models separately for 4 target variables relating to stimulus properties: visual location (VisL ≠ VisR), auditory location (AudL ≠ AudR), and spatial congruence at small (Incong5 ≠ Cong5) and large (Incong15 ≠ Cong15) disparities.

In both age groups, log model evidence summed over participants was greater for the [O>Y] than for the [O∩Y] set for all target variables. This suggests that the regions in which older participants show greater activations encode stimulus-relevant information better than the regions commonly activated in both age groups. Indeed, as shown in Fig 4, the age-related activation increases are found particularly in planum temporale and parietal cortices, which have previously been shown to be critical for encoding spatial information about auditory and visual stimuli and their spatial congruency [10,43,50].

**Fig 5:**
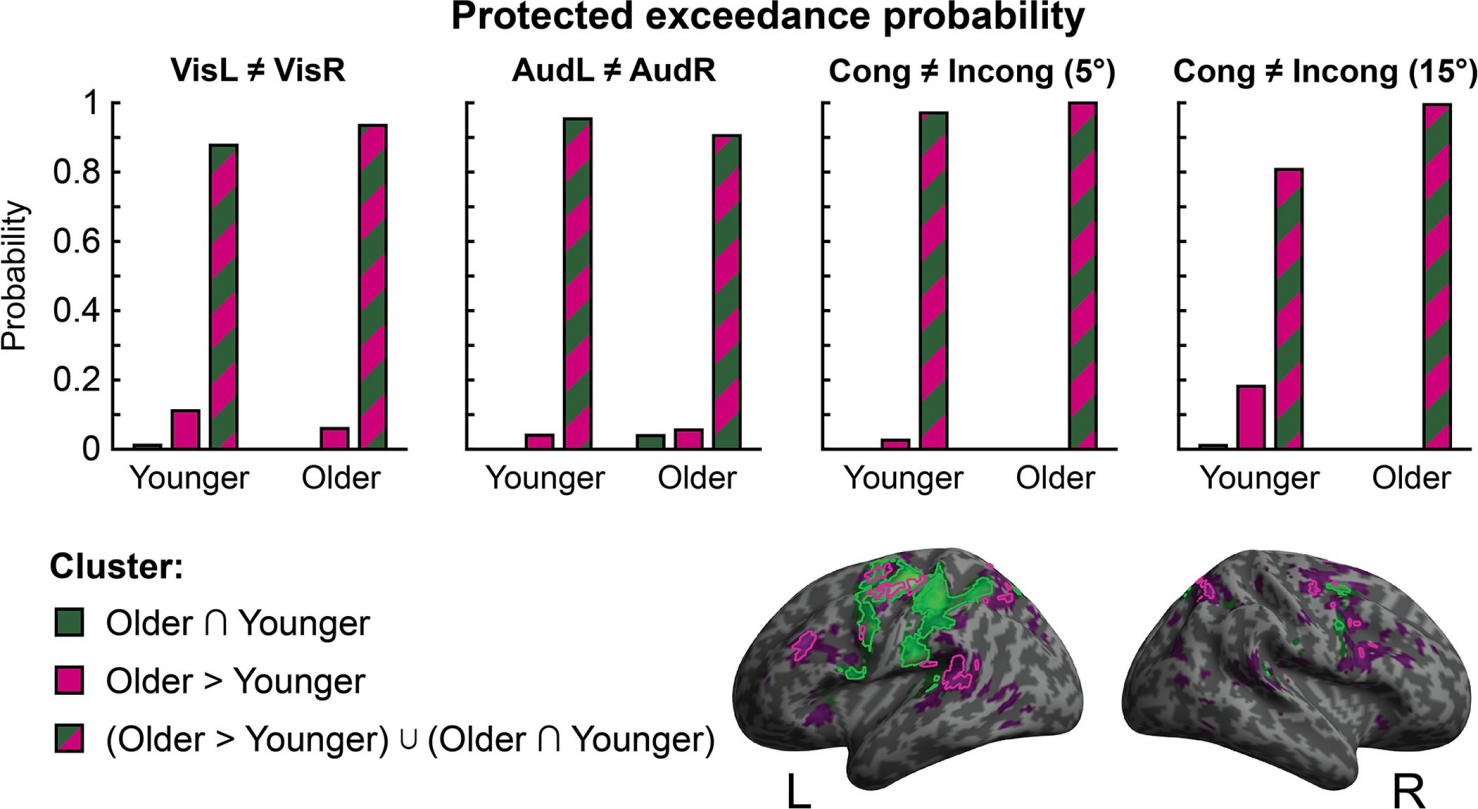
Results of multivariate Bayesian decoding analysis. Comparison of 3 sets of regions ([O∩Y], [O>Y], or union of both: [O>Y] ∪ [O∩Y]) in their ability to predict stimulus-related target variables: visual location, auditory location, congruent/incongruent at 5°, and congruent/incongruent at 15°. Protected exceedance probabilities, based on Bayesian model selection, are shown for each set of regions and target variable. The data underlying this Figure can be found in S1 Data.

Moreover, the union model [O>Y] ∪ [O∩Y] outperformed the more parsimonious models [O∩Y] and [O>Y] for each of the target variables. Bayesian model selection indicated that the protected exceedance probability was above 0.81 for the union model across all target variables in both age groups (see Fig 5). These model comparison results collectively show that, in both age groups, the regions with greater activations in older adults [O>Y] encode significant information about task-relevant variables that is complementary to the information encoded in regions commonly activated by younger and older adults [O∩Y].

Next, we asked whether this increase in stimulus and task-relevant information for [O>Y] regions is more prevalent or important in older adults, as they show more activations in these regions. To address this question, we assessed whether the union [O>Y] ∪ [O∩Y] relative to the more parsimonious models [O∩Y] and [O>Y] won more frequently in the older age group. Contrary to this conjecture, there were no significant age differences in the frequency with which the union model was the winning model for predicting any of the 4 target variables (χ^2^ tests of association, *p* > .05, *BF*_01_ ≥ 1.98).

To further explore possible age differences, we investigated the relative contributions of the 3 sets of regions to the encoding of task-relevant variables in older and younger participants. We did this by entering the difference in log model evidence for the union [O>Y] ∪ [O∩Y] set relative to the O∩Y set for each older and younger participant into Mann–Whitney U tests, separately for each of the 4 target variables. After Bonferroni correction for multiple comparisons, none of these tests revealed any significant differences between age groups across the VisL ≠ VisR (*U* = 116.000, *p* > .99, *BF*_01_ = 2.415, one tailed), AudL ≠ AudR (*U* = 126.000, *p* > .99, *BF*_01_ = 2.866, one tailed), and Incong5 ≠ Cong5 (*U* = 139.000, *p* > .99, *BF*_01_ = 2.568, one tailed) target variables (please note that Bayes factors do not contain any adjustment for multiple comparisons). Only for the Incong15 ≠ Cong15 target variable did we observe a small, nonsignificant trend for a greater “boost” in model evidence for the union [O>Y] ∪ [O∩Y] set, relative to the O∩Y set, for older adults compared to younger adults, *U* = 69.000, *p* = .052, *BF*_01_ = 0.616, one tailed.

Taken together, these results suggest that task-relevant information is encoded in each of the sets of regions and, in particular, in areas that are more strongly activated by older adults [O>Y], suggesting that older adults boost activations in brain regions that are critical for task-performance and encoding stimulus-relevant information. Further, the information encoded in the conjunction [O∩Y] and the “greater activation” [O>Y] sets were not redundant but at least partly complementary, so that the union set [O>Y] ∪ [O∩Y] outperformed both of those more parsimonious models. In other words, activation patterns in [O∩Y] and in [O>Y] made complementary contributions to encoding task- and stimulus-relevant variables.

Crucially, however, this was true for both older and younger adults. Likewise, the additional information gained by adding the “greater activation” [O>Y] set to the conjunction [O∩Y] set was comparable in both age groups. These results suggest that older adults show increased activations in brain areas that are important for encoding stimulus- and task-relevant information.

## Discussion

Healthy ageing leads to deficits in sensory processing and higher-order cognitive mechanisms. Nevertheless, older adults have been shown to maintain the ability to appropriately integrate and segregate audiovisual signals to aid stimulus localisation [32,51]. The present study investigated the neural mechanisms that support this maintenance of performance.

In agreement with previous research [20,32,51,52], our behavioural results suggest that older adults were largely able to maintain audiovisual spatial localisation accuracy. The responses of both age groups were consistent with the principles of Bayesian causal inference: Crossmodal biases were strongest when the sound and visual signals were spatially close together (and therefore more likely to share a common source), and weakest when the 2 signals were highly spatially separated (and therefore less likely to share a common source). We observed one small but significant three-way interaction between age, eccentricity, and sensory context. The profile of results (see Fig 1C) suggests that this effect was driven primarily by older adults’ sound localisation responses being more biased towards an incongruent visual stimulus (i.e., a greater ventriloquist effect) at large (30°) spatial disparities. These stronger audiovisual spatial biases for older adults at large spatial disparities were not observed in our previous behavioural research that took place outside the scanner [32]. One possibility is that they result from the greater attentional resources needed to effectively integrate or segregate audiovisual signals in the noisy environment of the MRI scanner. Background noise reduces a target sound’s signal-to-noise ratio, increasing the attentional resources required to identify and locate it, particularly in the presence of a highly salient and incongruent visual distractor (as in our large audiovisual disparity condition). As argued in a recent review [31], the greatest effects of ageing on multisensory integration are often found in situations of high attentional demand featuring, for example, noise or distractor signals (see, for instance, [53–55]). Similarly, small age-related hearing deficits may only become apparent under adverse listening conditions [56]. However, a similar result—older adults exhibiting stronger ventriloquist effects at larger spatial disparities—has previously been found even in the absence of background noise [33]. It is therefore possible that, rather than experimental design or stimulus factors, this small discrepancy in findings between our previous behavioural work [32] and the present study may be explained by differences in the samples. Perhaps the older participants in our behavioural study were simply less affected by age-related hearing loss or temporal processing deficits [39]. Future behavioural research could further explore these issues by systematically assessing the effects of ageing on spatial localisation in a ventriloquist task under various degrees of background noise, attentional load, and task demands in a large, diverse sample. It is also interesting to note that this behavioural effect is not reflected in the spatial representations decoded along the audiovisual processing hierarchy (discussed in more detail below), possibly because age-related differences arise in cortical areas beyond our regions of interest. However, given the differences between the fMRI and behavioural data and their analyses, it would be inappropriate to draw any strong conclusions here.

Having established that older and younger adults similarly integrate audiovisual signals into spatial perceptual reports, we next investigated their underlying neural representations as decoded from fMRI BOLD response patterns along the auditory and visual spatial processing pathways. As previously shown in human neuroimaging and neurophysiology studies [10,13,14,57–59], audiovisual interactions increased progressively across the cortical hierarchy. Primary auditory cortices (A1) encoded primarily the location of the auditory component of the stimuli, and early visual cortices (V1-V3) mainly that of the visual component, but small significant effects of sensory context and even audiovisual spatial congruency were observed even in primary visual areas. Again, these findings align nicely with a wealth of studies showing audiovisual interaction effects in primary sensory cortices [49,60–63]. Interestingly, a displaced visual stimulus biased the spatial encoding mainly at *small* spatial disparities in planum temporale, thereby mirroring the profile of crossmodal biases observed at the behavioural level that are consistent with Bayesian causal inference. By contrast, a displaced auditory stimulus biased the spatial encoding mainly at *large* spatial disparities in visual cortices. The latter suggests that the crossmodal biases on spatial representations decoded from visual cortices arise mainly from top-down, possibly attentional, influences. At small spatial disparities the perceived location of the less spatially reliable sound is shifted towards the visual location and thus does not affect spatial encoding in visual cortices. At large spatial disparities, audiovisual integration is attenuated or even abolished, so a spatially displaced sound may exert top-down attentional influences on the activation patterns in visual cortices.

Critically, none of these effects varied with age. Fig 2 shows that the decoded stimulus locations (averaged across participants) were near identical in older and younger adults for unisensory auditory, congruent audiovisual, and incongruent audiovisual stimuli in all ROIs. These results suggest that healthy ageing does not substantially alter how the brain integrates audiovisual inputs into spatial representations along the auditory or visual cortical pathways.

Despite these remarkably similar decoding profiles between the 2 age groups, across the auditory and visual processing hierarchies, we observed significantly greater BOLD responses across an extensive network of frontal, temporal, and parietal regions for older relative to younger adults in the spatial localisation task. This is in line with previous work showing age-related activation increases, especially in frontal and parietal regions, in a wide variety of situations [35,37,38,64,65], including those that involve processing of complex multisensory stimuli [66]. In the present study, older adults showed greater activations in areas such as superior temporal cortices (including plana temporalia), as well as inferior frontal sulci and intraparietal sulci. Some of these areas were adjacent to, or even partly overlapped with, those activated by both age groups (i.e., task-relevant activations above baseline were present in both groups but were greater in older adults).

This dissociation between age-related increases in regional BOLD responses, and comparable neural spatial representations along the audiovisual pathways, raises the question of what these activation increases contribute to task performance. What is their functional role? Specifically, we aimed to distinguish between 2 possible mechanisms: First, older adults may recruit additional areas to compensate for processing and representational encoding deficits in other regions. This idea has previously been suggested for a variety of scenarios in which older adults also showed increased activations [35,67,68] (though see also [37,69]). In such a case, we would expect that regions with age-related activation increases encode information about task-relevant variables more strongly in older than in younger adults.

Second, the age-related activation increases may not indicate compensatory recruitment of extra neural systems to encode stimulus- or task-relevant variables, but rather reflect more nonspecific processes. For instance, age-related activation increases may result from attentional or cognitive control mechanisms that are needed to form neural representations and produce behavioural responses that are matched in spatial precision and accuracy to their younger counterparts. Older adults may also increase activations to overcome inefficient neural processing or need more processing time to accumulate noisier evidence into spatial decisions, resulting in greater BOLD responses. Common to all these nonspecific mechanisms is that the set of regions exhibiting age-related activation increases should contribute similarly to encoding task-relevant information in older and younger populations.

To adjudicate between these 2 classes of neural mechanisms, we applied multivariate Bayesian decoding to compare the information about stimulus location and audiovisual congruency that is encoded in areas with (1) joint activations in both age groups [O∩Y], (2) increased activations in older adults [O>Y], and (3) the union of those 2 sets of regions [O>Y] ∪ [O∩Y]. All 3 sets of regions encoded task-relevant information about sound location and audiovisual spatial disparity. Moreover, formal model comparison indicated that the union model outperformed both of the more parsimonious models that included only 1 set of regions. This increase in model evidence for the union model indicates that regions with age-related activation increases [O>Y] and conjunction regions [O∩Y] provide complementary, rather than redundant, information about task-relevant variables. Further, it suggests that this information is encoded in a widespread, distributed way. Crucially, however, the boost in explanatory power when the regions were combined was comparable between younger and older adults.

Collectively, these results strongly argue against our first hypothesis that older adults engage new compensatory regions to encode stimulus variables. Instead, they align perfectly with previous work by Morcom and Henson [37], who also found that regions with age-related activation increases during memory tasks did not encode extra information in older adults. Likewise, Knights and colleagues [38] report that greater or more widespread activations in older adults did not encode more task-relevant information in a simple target detection/motor response task. Our results thus add to a growing body of research showing that age-related increases in BOLD activity are not indicative of “compensation by reorganisation” [70].

Together with this previous research, our multivariate Bayesian decoding results suggest that the activation increases may reflect more nonspecific compensatory processes. For example, our older adults may have expended more effort or top-down attentional control, used inefficient encoding strategies [38], or accumulated noisier sensory evidence for longer, to maintain spatial localisation performance despite age-related hearing loss or temporal processing deficits that make sound localisation more challenging. This would result in greater and more dispersed BOLD responses in key regions and is consistent with recent computational modelling of audiovisual spatial localisation in younger and older adults [32]. To differentiate between some of these potential mechanisms, future research may employ imaging methods with higher temporal resolution (such as magnetoencephalography) alongside stimuli with longer durations to compare the accumulation of sensory evidence over time between age groups [49]. Another possibility is that these age effects are related to general declines in γ-aminobutyric acid [71], which may lead to greater and less focused activations in older adults; this hypotheses would be a good future target for research employing magnetic resonance spectroscopy.

In conclusion, older adults show greater frontoparietal activations than their younger counterparts during audiovisual spatial integration. Yet, despite differences in BOLD response magnitude, the stimulus-relevant information encoded in these regions is comparable across the 2 age groups. Representations of audiovisual spatial stimuli in regions of the established dorsal auditory and visual processing pathways also remain remarkably unchanged in older adults. This dissociation—between comparable response accuracy and information encoded in brain activity patterns across the 2 age groups, but age-related activation increases—argues against the notion of “compensation by reorganisation” where new regions are recruited to encode stimulus- or task-relevant variables. Instead, our results suggest that age-related activation increases may reflect nonspecific mechanisms such as greater demands on attentional or cognitive control, or longer, less efficient, noisier neural encoding.

## Materials and methods

### Participants

Twenty younger and 29 older adults were initially recruited from participant databases for a behavioural screening session (see Materials and Methods in S1 Text for details). Two older adults were excluded from the study due to the presence of MRI contraindications, 3 failed to score above 24 on the Montreal Cognitive Assessment [72], and 1 reported taking antidepressant medication. A further 7 older, and 3 younger, adults were excluded for insufficient gaze fixation in the behavioural task. One younger participant could not be contacted following the behavioural session. Therefore, 16 younger (mean age = 24.19, *SD* = 4.56, 10 female) and 16 older (mean age = 70.75, *SD* = 4.71, 12 female) adults took part in all 3 experimental sessions. Those 32 included participants that had normal or corrected-to-normal vision, reported no hearing impairment, and were able to distinguish left from right sounds with a just-noticeable difference (JND) of below 10°. The study was approved by the University of Birmingham Ethical Review Committee (Application ERN_15-1458AP1). All participants gave informed consent and were compensated for their time in cash or research credits.

### Design and procedure (spatial ventriloquist paradigm inside the scanner)

In a spatial ventriloquist paradigm, participants were presented with synchronous auditory and visual signals at the same or different locations. The auditory signal originated from one of 4 possible spatial locations (−15°, −5°, 5°, or 15° visual angle) along the azimuth. For any given auditory location, a synchronous visual signal was presented at the same spatial location (audiovisual congruent trial), at the symmetrically opposite location (audiovisual incongruent trial), or was absent (unisensory auditory trial). On each trial, observers reported the sound location as accurately as possible by pressing one of 4 spatially corresponding buttons with their right hand. Thus, our design conformed to a 4 (auditory location: −15°, −5°, 5°, or 15° azimuth) × 3 (sensory context: unisensory auditory, audiovisual congruent, audiovisual incongruent) factorial design (see Fig 1B). Participants fixated a central cross (white; 0.75° diameter) throughout the experiment. Trials were presented with a stimulus onset asynchrony (SOA) of 2.3 seconds. To increase design efficiency, the activation trials were presented in a pseudorandomised fashion interleaved with 6.9-second fixation periods approximately every 20 trials. The experiment included 10 trials (per condition, per run) × 12 conditions × 11 five-minute runs (split over 2 separate days).

### Experimental setup

Stimuli were presented using Version 3 of the Psychophysics Toolbox [73], running on MATLAB 2014b on an Apple MacBook. Auditory stimuli were presented at approximately 75 dB SPL through Optime 1 electrodynamic headphones (MR Confon). Visual stimuli were back-projected by a JVC DLA-SX21E projector onto an acrylic screen, viewed via a mirror attached to the MRI head coil. The total viewing distance from eye to screen was 68 cm. Participants responded using infrared response pads (Nata Technologies) held in the right hand.

### Stimuli

Visual stimuli consisted of an 80-ms flash of 20 white dots (diameter of 0.4° visual angle), whose locations were sampled from a bivariate Gaussian distribution with a standard deviation of 2.5° in horizontal and vertical directions, presented on a black background.

Auditory spatialised stimuli (80 ms duration) were created by convolving a burst of white noise (with 5 ms onset and offset ramps) with spatially specific head-related transfer functions (HRTFs) based on the KEMAR dummy head of the MIT Media Lab [74]. Sounds were generated independently for every trial and presented with a 5-ms on/off ramp.

### Analysis of behavioural data (spatial ventriloquist paradigm inside the scanner)

For each participant, we calculated the mean auditory localisation response for each combination of auditory and visual locations. Responses to stimuli in the left hemifield were multiplied by −1, then participant-specific mean auditory localisation responses were entered into a 2 (hemifield: left or right) × 2 (eccentricity: 5° or 15°) × 3 (sensory context: unisensory auditory, audiovisual congruent, or audiovisual incongruent) × 2 (age group: younger or older) mixed ANOVA with the group factor as the only between-participants factor. An equivalent Bayesian mixed ANOVA, as implemented in JASP Version 0.16.4 [75], was also conducted, and result tables include *BF*_excl_ values for all main and interaction effects. These values represent the probability of the observed data occurring under a model that *excludes* a given term, relative to all other models. Thus, a higher number indicates more evidence that the term does not have predictive value within the model. JASP default priors were used for all Bayesian statistical tests. Analyses and underlying data, including of reaction times and participant responses during the behavioural screening session (which were substantively similar to responses inside the scanner), are all available in the Supporting information: see S1 Data for underlying data, and Fig A and Tables B-D in S1 Text for analyses.

Please note that many of the dependent variables analysed in this study are unlikely to be drawn from normal distributions. Though *t* tests and ANOVAs can be quite robust to this violation of their assumptions, individual analyses should be interpreted with caution (and considered in the context of the other information provided, such as descriptive plots and corresponding Bayesian tests).

### MRI data acquisition

A 3T Philips MRI scanner with a 32-channel head coil was used to acquire both T1-weighted anatomical images (TR = 8.4 ms, TE = 3.8 ms, flip angle = 8°, FOV = 288 mm × 232 mm, image matrix = 288 × 232, 175 sagittal slices acquired in ascending direction, voxel size = 1 × 1 × 1 mm) and T2*-weighted axial echoplanar images with bold oxygenation level-dependent (BOLD) contrast (gradient echo, SENSE factor of 2, TR = 2,800 ms, TE = 40 ms, flip angle = 90°, FOV = 192 mm × 192 mm, image matrix 76 × 76, 38 transversal slices acquired in ascending direction, voxel size = 2.5 × 2.5 × 2.5 mm with a 0.5-mm interslice gap).

Each participant took part in 2 one-hour scanning sessions, performed on separate days. In total (pooled over the 2 days), 11 task runs of 115 volumes each were acquired (i.e., 1,265 scanning volumes in total). Each scanning session also involved a further 115-volume resting-state run, during which participants were instructed to fixate a central cross. Four additional volumes were discarded from each scanning run prior to the analysis to allow for T_1_ equilibration effects.

### fMRI data analysis

Our fMRI analysis assessed the commonalities and differences in audiovisual spatial processing and integration between younger and older adults by combining 3 complementary methodological approaches. First, we used multivariate pattern decoding with support vector regression to characterise how auditory and visual information are combined into spatial representations along the dorsal visual and auditory processing hierarchies in younger and older participants. Second, we used conventional mass-univariate analyses to investigate how congruent and incongruent audiovisual stimulation influences univariate BOLD responses across the entire brain. Third, we used multivariate Bayesian decoding to assess how the neural systems that show greater activations for older adults, as well as those that were activated in both groups, encode information about the spatial location or congruency of audiovisual stimuli.

#### Preprocessing and within-participant (first-level) general linear models

MRI data were analysed in SPM12 [76]. Each participant’s functional scans were realigned/unwarped to correct for movement, slice-time corrected, and coregistered to the anatomical scan. For multivariate pattern decoding (i.e., support vector regression and multivariate Bayesian decoding), these native-space data were spatially smoothed with a Gaussian kernel of 3 mm FWHM. For mass-univariate analyses and multivariate Bayesian decoding, the slice-time-corrected and realigned images were normalised into Montreal Neurological Institute (MNI) space using parameters from segmentation of the T1 structural image [77], resampled to a spatial resolution of 2 × 2 × 2 mm^3^ and spatially smoothed with a Gaussian kernel of 8 mm full-width at half-maximum.

The following processing steps were conducted separately on both native-space and MNI-transformed data. Each voxel’s time series was high-pass filtered to 1/128 Hz. The fMRI experiment was modelled in an event-related fashion with regressors entered into the design matrix after convolving each event-related unit impulse (coding the stimulus onset) with a canonical hemodynamic response function and its first temporal derivative. In addition to modelling the 12 conditions in our 4 (auditory location: −15°, −5°, 5°, or 15° visual angle) × 3 (sensory context: unisensory auditory, audiovisual congruent, audiovisual incongruent) within-participant factorial design, the model included the realignment parameters as nuisance covariates to account for residual motion artifacts. For the mass-univariate analysis and the multivariate Bayesian decoding analysis, the design matrix also modelled the button response choices as a single regressor to account for motor responses. To enable more reliable estimates of the activation patterns, we did not account for observers’ response choices in the support vector regression analysis that is reported in this manuscript (sound locations and observers’ sound localisation responses were highly correlated). However, a control analysis confirmed that the fMRI decoded spatial locations did not differ across age groups when observers’ spatially specific responses were also modelled.

#### Correcting BOLD response for age-related changes in vascular reactivity

The normal ageing process can lead to complex and nonuniform changes in vascular reactivity and neurovascular coupling [78,79]. To at least partly account for these changes, we corrected the BOLD-response amplitude (i.e., parameter estimates pertaining to the canonical hemodynamic response function) in each voxel in the MNI-normalised data based on the resting state fluctuation amplitude (or scan-to-scan signal variability) [79,80]. Resting-state data were preprocessed exactly as the task (i.e., spatial ventriloquist) data (i.e., realigned/unwarped, slice-time corrected, coregistered to the anatomical image, normalised to MNI space, resampled, and spatially smoothed with a Gaussian kernel of 8 mm FWHM). We applied additional steps to minimise the effect of motion, and other nuisance variables, on the signal. First, we applied wavelet despiking [81] and linear and quadratic detrending. The BOLD response over scans was then residualised with respect to the following regressors: white matter signal (the mean across all voxels containing white matter, according to SPM’s automated segmentation algorithm, was taken for each volume, and the time-varying signal included as a regressor); cerebrospinal fluid signal (using the same procedure as with white matter); and movement parameters (and their first derivatives). The signal was then bandpass-filtered at 0.01 to 0.08 Hz to maximise the contribution of physiological factors to the signal fluctuation. The standard deviation of the remaining variation across scans at each voxel was calculated to create the final resting state fluctuation map (separately for each scanning day). The parameter estimates in each voxel, condition, and participant were standardised by dividing by the relevant resting state fluctuation amplitude value prior to further analysis.

#### Decoding audiovisual spatial representations using support vector regression

Using multivariate pattern decoding with support vector regression, we investigated how younger and older adults combine auditory and visual signals into spatial representations along the auditory and visual processing hierarchies. The basic rationale of this analysis is as follows: We first train a model to learn the mapping from fMRI activation patterns in ROIs to stimulus locations in the external world based solely on congruent audiovisual stimuli. We then use this learnt mapping to decode the spatial locations from activation patterns of the incongruent audiovisual signals. In putatively unisensory auditory regions, locations decoded from fMRI activation patterns for incongruent trials should therefore reflect only the sound location (irrespective of the visual location); in unisensory visual regions, decoded locations should reflect only the visual location; and in audiovisual integration regions, the decoded locations should be somewhere between the auditory and visual locations. Hence, the locations decoded from activation patterns for audiovisual incongruent stimuli provide insights into how regions weigh and combine spatial information from vision and audition. This approach is closely linked to our behavioural analysis, which focuses on how observers weight and combine audiovisual signals into spatial percepts or reported locations.

For the multivariate decoding analysis, we extracted the parameter estimates of the canonical hemodynamic response function for each condition and run from voxels of the regions of interest (i.e., fMRI activation vectors; see ROI section below). The parameter estimates pertaining to the canonical hemodynamic response function defined the magnitude of the BOLD response to the auditory and audiovisual stimuli in each voxel. Each fMRI activation vector for the 12 conditions in our 4 (auditory location) × 3 (sensory context) factorial design was based on 10 trials within a particular run. Activation vectors were normalised to between 0 and 1.

For each of the 5 ROIs along the visual and auditory processing hierarchies, we trained a support vector regression model (with default parameters *C* = 1 and γ = 1/n features, as implemented in LIBSVM 3.17 [82], accessed via The Decoding Toolbox Version 3.96 [83]) to learn the mapping from the fMRI activation vectors to the external spatial locations based on the audiovisual spatially *congruent* conditions from all but one of the 11 runs. This learnt mapping from activation patterns to external spatial locations was then used to decode the spatial location from the fMRI activation patterns of the unisensory auditory, audiovisual congruent, and audiovisual incongruent conditions of the remaining run. In a leave-one-run-out cross-validation scheme, the training-test procedure was repeated for all 11 runs. The decoded spatial estimates for each condition were then averaged across runs.

The decoded spatial estimates were then analysed in the same way as the behavioural data: Responses to stimuli in the left hemifield were multiplied by −1, then condition-specific estimates were entered into a 2 (hemifield: left or right) × 2 (eccentricity: 5° or 15°) × 3 (sensory context: unisensory auditory, audiovisual congruent, or audiovisual incongruent) × 2 (age group: younger or older) mixed ANOVA at the second (random effects) level separately for each ROI. For analysis, incongruent conditions were labelled based on the location of the stimulus that corresponds with the ROI’s dominant sensory modality: V1-V3 and intraparietal sulcus responses were labelled based on the location of the visual stimulus; planum temporale and A1 were labelled based on the location of the auditory stimulus. As with the behavioural data, corresponding Bayesian mixed ANOVAs [75] were also conducted, and results tables include *BF*_excl_ values for all main and interaction effects. Versions of the analyses where all incongruent stimuli were labelled based on the auditory location are also available in Tables K-M in S1 Text, though note that this approach introduces artificial interaction effects between stimulus eccentricity and audiovisual congruence for visual-dominant ROIs.

*Regions of interest for support vector regression analysis.* Our support vector regression analysis selectively focused on regions along the dorsal auditory and visual spatial processing pathways that have previously been shown to be critical for integrating auditory and visual signals into spatial representations [5,10,13,14,61]. Specifically, we defined 5 ROIs based on inverse-normalised group-level probabilistic maps. Left and right hemisphere maps were combined. Visual (V1-V3) and intraparietal sulcus (IPS 0–2, IPS 3–4) ROIs were defined using retinotopic maximum probability maps [44]. Primary auditory cortex (A1) was defined based on cytoarchitectonic maximum probability maps [84]. Planum temporale was defined based on labels of the Destrieux atlas [85,86], as implemented in Freesurfer 5.3.0 [87].

#### Conventional second-level mass-univariate analysis: Identifying stimulus- and task-related activations

Using conventional mass-univariate analysis, we next characterised activations for audiovisual stimuli relative to fixation, and audiovisual spatial incongruence, across the entire brain, and compared between older and younger participants. At the first level, condition-specific effects for each participant were estimated according to the general linear model (see earlier section) and passed to a second-level ANOVA as contrasts. Inferences were made at the second level to allow for random effects analysis and population-level inferences [88].

At the random effects (i.e., group) level, we tested for:

1. Effects present in both age groups for all stimuli (unisensory auditory, audiovisual congruent, and audiovisual incongruent) relative to fixation:
  - (All_Older_ > Fixation_Older_) ∩ (All_Younger_ > Fixation_Younger_)
2. Age group differences in the effects of all stimuli relative to fixation:
  - (All_Older_ > Fixation_Older_) > (All_Younger_ > Fixation_Younger_)
  - (All_Younger_ > Fixation_Younger_) > (All_Older_ > Fixation_Older_)
3. The effect of audiovisual spatial incongruence, averaged across age groups:
  - Incong > Cong
4. The interaction between audiovisual spatial incongruence and age group:
  - (Incong_Older_ > Cong_Older_) > (Incong_Younger_ > Cong_Younger_)
  - (Incong_Younger_ > Cong_Younger_) > (Incong_Older_ > Cong_Older_)

Unless otherwise stated, activations are reported at *p* < .05 at the voxel level, familywise error corrected for multiple comparisons across the entire brain.

#### Multivariate Bayesian decoding to compare the ability of sets of regions to predict task-relevant variables

We assessed the extent to which activations identified by the mass-univariate analysis contributed to encoding of visual or auditory location, and their spatial relationship (i.e., congruence), in younger and older participants. Our key question was whether regions with greater activations for older than younger adults contribute more to encoding these task-relevant variables in both age groups.

To address this question, we used multivariate Bayesian decoding, as implemented in SPM12 [42], which estimates the set of activation patterns that best predicts a particular target variable such as visual or auditory location using hierarchical parametric empirical Bayes. Multivariate Bayes treats a set of regions as a model for encoding a particular target variable (for instance, auditory location left versus right). It estimates the log model evidence, which trades off model accuracy with complexity [42,89]. The model evidence can then be used to compare different models using Bayesian model selection (BMS) at the group (i.e., random effects) level [90]. Hence, unlike support vector regression, multivariate Bayesian decoding allows us to compare the relative contributions of different areas of interest to encoding or predicting a particular target variable (for instance, auditory location left versus right) using standard procedures of Bayesian model comparison. Specifically, we used multivariate Bayesian decoding to compare the contributions of 3 functionally defined sets of regions to encoding stimulus and task-relevant variables:

1. Activations that are common to younger and older participants (referred to as [O∩Y]), as specified by the conjunction (using the conjunction null [46,47]): *(All*_*Older*_ *> Fixation*_*Older*_*) ∩ (All*_*Younger*_ *> Fixation*_*Younger*_*)*.
2. Activations that were enhanced for older relative to younger participants (referred to as [O>Y]), as specified by: *(All*_*Older*_ *> Fixation*_*Older*_*) > (All*_*Younger*_ *> Fixation*_*Younger*_*)*.
3. The union [O>Y] ∪ [O∩Y] of each of the above 2 sets of regions.

These sets of regions were defined based on the respective inverse normalised statistical comparisons at the random effects group level, using a leave-one-participant-out scheme. They were constrained to include only the 1,000 voxels with the greatest *t* value for the respective comparisons; the union set [O>Y] ∪ [O∩Y] was created by randomly sampling 500 unique (nonoverlapping) voxels from each of the 2 component sets of regions.

For each set of regions, we fitted 4 independent multivariate Bayes models, predicting different target variables:

1. Visual location [VisL ≠ VisR]
2. Auditory location [AudL ≠ AudR]
3. Incongruence with 5° eccentricity [Incong5 ≠ Cong5]
4. Incongruence with 15° eccentricity [Incong15 ≠ Cong15]

Both predictor and target variables were residualised with respect to effects of no interest (i.e., all general linear model covariates other than those involved in the target contrast).

Please note that the contrasts used to define sets of regions were orthogonal to the target variables (for instance, the contrast [All > Fixation], pooled over both age groups, is orthogonal to visual location [VisL ≠ VisR]). Moreover, the sets of regions were defined using a leave-one-participant-out cross-validation scheme, so each participant’s own activations were not used to define their participant-specific sets.

Separate multivariate Bayes models were fitted for each participant, for each set of regions, and for each target variable. We entered the resulting log model evidence values into statistical analyses and Bayesian model comparison procedures to assess the contributions of the 3 different sets of regions to the encoding of the 4 target variables and to explore whether/how these contributions varied with age. More specifically, the analysis included the following steps:

First, we assessed whether information is encoded in a more sparse or distributed fashion in each region by comparing models in which patterns are individual voxels (i.e., “sparse”) versus clusters (i.e., smooth spatial prior). In our data, the sparse model (in which the weights of individual voxels are optimised) outperformed the smooth model across all analyses (paired-sample *t* tests of log model evidences, *p <* .001), so we will focus selectively on the results from this model class.

We also ensured that the target variables could be decoded reliably from each set of regions by comparing the evidence for each “model of interest” with the evidence of models in which the design matrix had been randomly phase shuffled (i.e., stimulus onset times uniformly shifted by a random amount; this was repeated 20 times, and the mean of the log model evidence was taken; see, for instance, [37] for a similar approach). Using *t* tests, we compared the difference in real versus shuffled model evidences and confirmed that the real models performed significantly better for all sets of regions and target variables (*p* < .05, one tailed) except Incong15 ≠ Cong15 in the O∩Y set of regions, *t*(31) = 1.24, *p* = .113.

Next, and more importantly, we assessed which of the 3 candidate sets of regions (i.e., (1) [O∩Y], the conjunction of activations in older and younger; (2) [O>Y], activation increases in older relative to younger adults; or (3) [O>Y] ∪ [O∩Y], the union of sets 1 and 2) is the best model or predictor for each of the target variables, separately for the older and younger groups, by performing Bayesian model selection at the random effects (group) level, as implemented in SPM12 [90]. We report log model evidence values, as well as the protected exceedance probability that a given model is better than any of the other candidate models beyond chance [91]. If the regions with greater activations in older (relative to younger) adults make critical contributions to encoding the task-relevant target variable, we would expect the model evidence for the union [O>Y] ∪ [O∩Y] to exceed that of the conjunction model [O∩Y]. Further, we formally assessed whether the frequency with which each model “won” differed between age groups using a χ^2^ test of association (1 test per target variable). We report *p* values after Bonferroni correction for multiple (i.e., 4 target variables) comparisons.

Finally, we investigated whether the set of regions with greater activations for older participants (i.e., [O>Y] set) contributes more to the encoding of the critical target variables in older adults by comparing the difference in log model evidence for the union [O>Y] ∪ [O∩Y] set relative to the joint [O∩Y] set between older and younger adults in a nonparametric Mann–Whitney U tests separately for each of the 4 target variables (VisL ≠ VisR, AudL ≠ AudR, Incong5 ≠ Cong5, and Incong15 ≠ Cong15). We report *p* values after Bonferroni correction for multiple (i.e., 4 target variables) comparisons. Full output from these tests, as well as corresponding Bayesian statistics [75], are available in Table N in S1 Text.

## Supporting information

S1 Data Excel spreadsheet with individual numerical data organised into separate sheets corresponding to the following: Figs 1C, 2A, 2B, 2C, 5, and ABCI in S1 Text; and Tables 1, 2, and A-N in S1 Text. (XLSX)

S2 Data ZIP file containing the second-level general linear model from the mass-univariate analysis, including values underlying the following: Figs 3, 4, and D-H in S1 Text; and Table 3. The data are stored in MATLAB structures and NIfTI files and are best viewed using the SPM12 toolbox.
(ZIP)

S1 Text PDF document containing supporting results and methods. (PDF)

S1 File Custom MATLAB code. (ZIP)

## Abbreviations

BOLD: blood-oxygen-level-dependent
fMRI: functional magnetic resonance imaging
MNI: Montreal Neurological Institute
ROI: region of interest
