# Supplementary material for: Older adults preserve audiovisual integration through enhanced cortical activations, not by recruiting new regions
Source: PLoS Biol. 2024 Feb 6;22(2):e3002494. doi: 10.1371/journal.pbio.3002494 (PMC10871488; doi:10.1371/journal.pbio.3002494)
Supplement: S1 Text — (PDF) [file pbio.3002494.s003.pdf]

Older adults preserve audiovisual integration through enhanced cortical activations, not by recruiting new regions: Supplementary results and methods

Results

Audiovisual integration behaviour

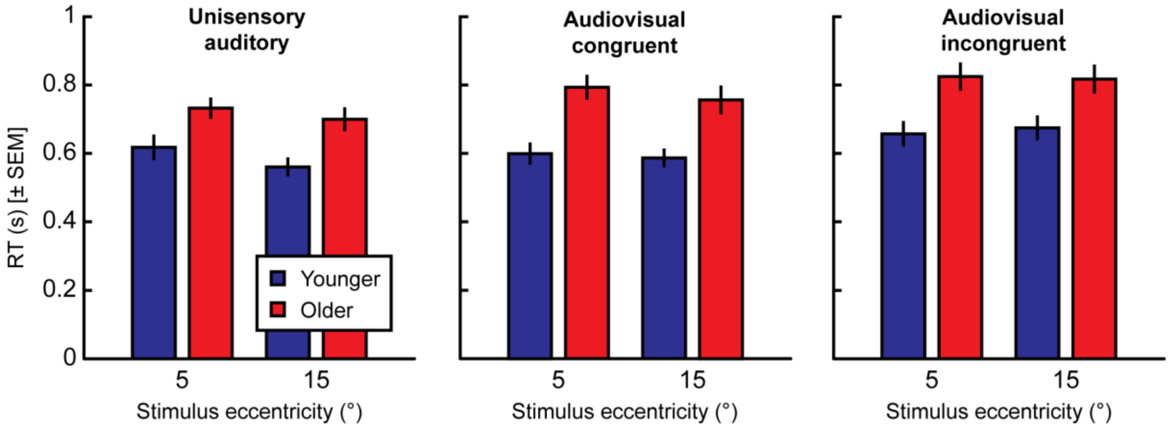

Fig A. Behavioural results (response times).

Behavioural response times (pooled over left and right hemifields; across-participants means of condition-specific medians). Participants responded more slowly to audiovisual incongruent relative to audiovisual congruent and auditory-only stimuli. Older adults were significantly slower in all conditions, but this did not interact with any other factor. The data underlying this Figure can be found in S1 Data.

Table A. Follow-up* t *tests for results of mixed ANOVA on localisation responses during the spatial ventriloquist task (inside the scanner).

Independent Samples T-Test

	t	df	p
L15_unimodal	1.280	30	0.210
L15_congruent	1.023	30	0.314
L15_incongruent	1.154	30	0.258
L5_unimodal	1.067	30	0.295
L5_congruent	−0.232	30	0.818
L5_incongruent	−0.188	30	0.852
R5_unimodal	1.441	30	0.160
R5_congruent	1.015	30	0.318
R5_incongruent	0.172	30	0.865
R15_unimodal	1.229	30	0.229
R15_congruent	0.035	30	0.973
R15_incongruent	1.783	30	0.085[a]

Note. Student's t-test.

[a] Brown-Forsythe test is significant ($p < .05$), suggesting a violation of the equal variance assumption

Bayesian Independent Samples T-Test

	BF_{01}	error %
L15_unimodal	1.598	0.004
L15_congruent	1.994	0.004
L15_incongruent	1.792	0.004
L5_unimodal	1.927	0.004
L5_congruent	2.913	0.003
L5_incongruent	2.934	0.003
R5_unimodal	1.357	0.004
R5_congruent	2.007	0.004
R5_incongruent	2.941	0.003
R15_unimodal	1.675	0.004
R15_congruent	2.973	0.003
R15_incongruent	0.907	0.005

Table B. Results of mixed ANOVA on median response times during the spatial ventriloquist task (inside the scanner).

Within Subjects Effects

Cases	Sphericity Correction	Sum of Squares	df	Mean Square	F	p	η^2_p
Hemifield	None	3.548e−5	1.000	3.548e−5	0.005	0.945	1.637e−4
Hemifield ∗ ageGroup	None	0.004	1.000	0.004	0.576	0.454	0.019
Residuals	None	0.217	30.000	0.007			
Eccentricity	None	0.042	1.000	0.042	3.120	0.088	0.094
Eccentricity ∗ ageGroup	None	0.002	1.000	0.002	0.113	0.739	0.004
Residuals	None	0.402	30.000	0.013			
Sensory Context	None	0.533	2.000	0.267	33.370	1.807e−10	0.527
	Greenhouse-Geisser	0.533	1.916	0.278	33.370	4.017e−10	0.527
Sensory Context ∗ ageGroup	None	0.041	2.000	0.020	2.559	0.086	0.079
	Greenhouse-Geisser	0.041	1.916	0.021	2.559	0.088	0.079
Residuals	None	0.479	60.000	0.008			
	Greenhouse-Geisser	0.479	57.486	0.008			
Hemifield ∗ Eccentricity	None	0.002	1.000	0.002	0.577	0.454	0.019
Hemifield ∗ Eccentricity ∗ ageGroup	None	0.002	1.000	0.002	0.674	0.418	0.022
Residuals	None	0.095	30.000	0.003			
Hemifield ∗ Sensory Context	None	0.018[a]	2.000[a]	0.009[a]	5.082[a]	0.009[a]	0.145
	Greenhouse-Geisser	0.018	1.477	0.012	5.082	0.017	0.145
Hemifield ∗ Sensory Context ∗ ageGroup	None	2.591e−5[a]	2.000[a]	1.296e−5[a]	0.007[a]	0.993[a]	2.391e−4
	Greenhouse-Geisser	2.591e−5	1.477	1.755e−5	0.007	0.977	2.391e−4
Residuals	None	0.108	60.000	0.002			
	Greenhouse-Geisser	0.108	44.309	0.002			
Eccentricity ∗ Sensory Context	None	0.034[a]	2.000[a]	0.017[a]	5.942[a]	0.004[a]	0.165
	Greenhouse-Geisser	0.034	1.565	0.022	5.942	0.009	0.165
Eccentricity ∗ Sensory Context ∗ ageGroup	None	0.010[a]	2.000[a]	0.005[a]	1.787[a]	0.176[a]	0.056
	Greenhouse-Geisser	0.010	1.565	0.006	1.787	0.185	0.056
Residuals	None	0.171	60.000	0.003			
	Greenhouse-Geisser	0.171	46.941	0.004			
Hemifield ∗ Eccentricity ∗ Sensory Context	None	6.579e−4[a]	2.000[a]	3.290e−4[a]	0.114[a]	0.892[a]	0.004
	Greenhouse-Geisser	6.579e−4	1.627	4.045e−4	0.114	0.852	0.004
Hemifield ∗ Eccentricity ∗ Sensory Context ∗ ageGroup	None	0.004[a]	2.000[a]	0.002[a]	0.774[a]	0.466[a]	0.025
	Greenhouse-Geisser	0.004	1.627	0.003	0.774	0.442	0.025
Residuals	None	0.173	60.000	0.003			
	Greenhouse-Geisser	0.173	48.796	0.004			

Note. Sphericity corrections not available for factors with 2 levels.
Note. Type III Sum of Squares
[a] Mauchly's test of sphericity indicates that the assumption of sphericity is violated (p < .05).

Between Subjects Effects ▼

Cases	Sum of Squares	df	Mean Square	F	p	η^2_p
ageGroup	2.322	1	2.322	11.000	0.002	0.268
Residuals	6.334	30	0.211			

Note. Type III Sum of Squares

Analysis of Effects

Effects	P(incl)	P(excl)	P(incl|data)	P(excl|data)	BF_{excl}
Hemifield	0.886	0.114	0.458	0.542	9.222
Eccentricity	0.886	0.114	0.915	0.085	0.722
Sensory Context	0.886	0.114	1.000	2.266e−9	1.765e−8
ageGroup	0.886	0.114	0.967	0.033	0.269
Hemifield ∗ Eccentricity	0.503	0.497	0.091	0.909	10.087
Hemifield ∗ Sensory Context	0.503	0.497	0.267	0.733	2.776
Eccentricity ∗ Sensory Context	0.503	0.497	0.788	0.212	0.272
Hemifield ∗ Eccentricity ∗ Sensory Context	0.120	0.880	0.005	0.995	27.755
Hemifield ∗ ageGroup	0.503	0.497	0.124	0.876	7.144
Eccentricity ∗ ageGroup	0.503	0.497	0.275	0.725	2.665
Hemifield ∗ Eccentricity ∗ ageGroup	0.120	0.880	0.003	0.997	43.560
Sensory Context ∗ ageGroup	0.503	0.497	0.508	0.492	0.979
Hemifield ∗ Sensory Context ∗ ageGroup	0.120	0.880	0.004	0.996	33.063
Eccentricity ∗ Sensory Context ∗ ageGroup	0.120	0.880	0.043	0.957	3.054
Hemifield ∗ Eccentricity ∗ Sensory Context ∗ ageGroup	0.006	0.994	1.195e−6	1.000	5041.814

Table C. Results of mixed ANOVA on localisation responses during the spatial ventriloquist task (screening session outside the scanner).

Within Subjects Effects

Cases	Sphericity Correction	Sum of Squares	df	Mean Square	F	p	η^2_p
Hemifield	None	96.690	1.000	96.690	4.447	0.043	0.129
Hemifield ✻ ageGroup	None	58.982	1.000	58.982	2.713	0.110	0.083
Residuals	None	652.307	30.000	21.744			
Eccentricity	None	4185.646	1.000	4185.646	250.085	4.268e−16	0.893
Eccentricity ✻ ageGroup	None	42.609	1.000	42.609	2.546	0.121	0.078
Residuals	None	502.107	30.000	16.737			
Sensory Context	None	3800.658[a]	2.000[a]	1900.329[a]	47.470[a]	4.362e−13[a]	0.613
	Greenhouse-Geisser	3800.658	1.146	3316.346	47.470	1.905e−8	0.613
Sensory Context ✻ ageGroup	None	90.776[a]	2.000[a]	45.388[a]	1.134[a]	0.329[a]	0.036
	Greenhouse-Geisser	90.776	1.146	79.209	1.134	0.303	0.036
Residuals	None	2401.956	60.000	40.033			
	Greenhouse-Geisser	2401.956	34.381	69.863			
Hemifield ✻ Eccentricity	None	0.768	1.000	0.768	0.133	0.718	0.004
Hemifield ✻ Eccentricity ✻ ageGroup	None	9.231	1.000	9.231	1.599	0.216	0.051
Residuals	None	173.227	30.000	5.774			
Hemifield ✻ Sensory Context	None	11.472	2.000	5.736	1.507	0.230	0.048
	Greenhouse-Geisser	11.472	1.885	6.087	1.507	0.231	0.048
Hemifield ✻ Sensory Context ✻ ageGroup	None	16.551	2.000	8.275	2.174	0.123	0.068
	Greenhouse-Geisser	16.551	1.885	8.782	2.174	0.126	0.068
Residuals	None	228.435	60.000	3.807			
	Greenhouse-Geisser	228.435	56.537	4.040			
Eccentricity ✻ Sensory Context	None	35.961[a]	2.000[a]	17.981[a]	1.280[a]	0.285[a]	0.041
	Greenhouse-Geisser	35.961	1.271	28.285	1.280	0.276	0.041
Eccentricity ✻ Sensory Context ✻ ageGroup	None	47.776[a]	2.000[a]	23.888[a]	1.701[a]	0.191[a]	0.054
	Greenhouse-Geisser	47.776	1.271	37.579	1.701	0.201	0.054
Residuals	None	842.582	60.000	14.043			
	Greenhouse-Geisser	842.582	38.141	22.091			
Hemifield ✻ Eccentricity ✻ Sensory Context	None	5.994	2.000	2.997	1.211	0.305	0.039
	Greenhouse-Geisser	5.994	1.725	3.475	1.211	0.301	0.039
Hemifield ✻ Eccentricity ✻ Sensory Context ✻ ageGroup	None	10.466	2.000	5.233	2.115	0.130	0.066
	Greenhouse-Geisser	10.466	1.725	6.068	2.115	0.137	0.066
Residuals	None	148.457	60.000	2.474			
	Greenhouse-Geisser	148.457	51.747	2.869			

Note. Sphericity corrections not available for factors with 2 levels.
Note. Type III Sum of Squares
[a] Mauchly's test of sphericity indicates that the assumption of sphericity is violated ($p < .05$).

Between Subjects Effects

Cases	Sum of Squares	df	Mean Square	F	p	η^2_p
ageGroup	165.366	1	165.366	3.531	0.070	0.105
Residuals	1404.847	30	46.828			

Note. Type III Sum of Squares

Analysis of Effects

Effects	P(incl)	P(excl)	P(incl|data)	P(excl|data)	BF_{excl}
Hemifield	0.886	0.114	0.816	0.184	1.761
Eccentricity	0.886	0.114	1.000	1.799e−14	1.401e−13
Sensory Context	0.886	0.114	1.000	2.189e−11	1.705e−10
ageGroup	0.886	0.114	0.883	0.117	1.029
Hemifield ✻ Eccentricity	0.503	0.497	0.184	0.816	4.488
Hemifield ✻ Sensory Context	0.503	0.497	0.177	0.823	4.707
Eccentricity ✻ Sensory Context	0.503	0.497	0.331	0.669	2.047
Hemifield ✻ Eccentricity ✻ Sensory Context	0.120	0.880	0.003	0.997	38.936
Hemifield ✻ ageGroup	0.503	0.497	0.367	0.633	1.744
Eccentricity ✻ ageGroup	0.503	0.497	0.466	0.534	1.160
Hemifield ✻ Eccentricity ✻ ageGroup	0.120	0.880	0.020	0.980	6.698
Sensory Context ✻ ageGroup	0.503	0.497	0.350	0.650	1.878
Hemifield ✻ Sensory Context ✻ ageGroup	0.120	0.880	0.018	0.982	7.431
Eccentricity ✻ Sensory Context ✻ ageGroup	0.120	0.880	0.035	0.965	3.755
Hemifield ✻ Eccentricity ✻ Sensory Context ✻ ageGroup	0.006	0.994	3.255e−5	1.000	185.055

Table D. Results of mixed ANOVA on median response times during the spatial ventriloquist task (screening session outside the scanner).

Within Subjects Effects

Cases	Sphericity Correction	Sum of Squares	df	Mean Square	F	p	η^2_p
Hemifield	None	0.014	1.000	0.014	0.863	0.360	0.028
Hemifield ✻ ageGroup	None	0.001	1.000	0.001	0.071	0.791	0.002
Residuals	None	0.476	30.000	0.016			
Eccentricity	None	0.356	1.000	0.356	23.831	3.253e−5	0.443
Eccentricity ✻ ageGroup	None	0.069	1.000	0.069	4.598	0.040	0.133
Residuals	None	0.448	30.000	0.015			
Sensory Context	None	1.204[a]	2.000[a]	0.602[a]	32.645[a]	2.553e−10[a]	0.521
	Greenhouse-Geisser	1.204	1.643	0.732	32.645	7.229e−9	0.521
Sensory Context ✻ ageGroup	None	0.045[a]	2.000[a]	0.023[a]	1.233[a]	0.299[a]	0.039
	Greenhouse-Geisser	0.045	1.643	0.028	1.233	0.294	0.039
Residuals	None	1.106	60.000	0.018			
	Greenhouse-Geisser	1.106	49.299	0.022			
Hemifield ✻ Eccentricity	None	8.853e−4	1.000	8.853e−4	0.116	0.736	0.004
Hemifield ✻ Eccentricity ✻ ageGroup	None	0.002	1.000	0.002	0.274	0.604	0.009
Residuals	None	0.228	30.000	0.008			
Hemifield ✻ Sensory Context	None	0.012	2.000	0.006	0.812	0.449	0.026
	Greenhouse-Geisser	0.012	1.852	0.007	0.812	0.441	0.026
Hemifield ✻ Sensory Context ✻ ageGroup	None	0.001	2.000	5.259e−4	0.069	0.934	0.002
	Greenhouse-Geisser	0.001	1.852	5.679e−4	0.069	0.922	0.002
Residuals	None	0.458	60.000	0.008			
	Greenhouse-Geisser	0.458	55.564	0.008			
Eccentricity ✻ Sensory Context	None	0.102[a]	2.000[a]	0.051[a]	8.648[a]	5.009e−4[a]	0.224
	Greenhouse-Geisser	0.102	1.495	0.068	8.648	0.002	0.224
Eccentricity ✻ Sensory Context ✻ ageGroup	None	0.028[a]	2.000[a]	0.014[a]	2.420[a]	0.098[a]	0.075
	Greenhouse-Geisser	0.028	1.495	0.019	2.420	0.114	0.075
Residuals	None	0.353	60.000	0.006			
	Greenhouse-Geisser	0.353	44.851	0.008			
Hemifield ✻ Eccentricity ✻ Sensory Context	None	0.019[a]	2.000[a]	0.009[a]	1.273[a]	0.287[a]	0.041
	Greenhouse-Geisser	0.019	1.480	0.013	1.273	0.282	0.041
Hemifield ✻ Eccentricity ✻ Sensory Context ✻ ageGroup	None	0.012[a]	2.000[a]	0.006[a]	0.803[a]	0.453[a]	0.026
	Greenhouse-Geisser	0.012	1.480	0.008	0.803	0.421	0.026
Residuals	None	0.440	60.000	0.007			
	Greenhouse-Geisser	0.440	44.407	0.010			

Note. Sphericity corrections not available for factors with 2 levels.
Note. Type III Sum of Squares
[a] Mauchly's test of sphericity indicates that the assumption of sphericity is violated (p < .05).

Between Subjects Effects ▼

Cases	Sum of Squares	df	Mean Square	F	p	η^2_p
ageGroup	3.687	1	3.687	16.392	3.340e−4	0.353
Residuals	6.749	30	0.225			

Note. Type III Sum of Squares

Analysis of Effects

Effects	P(incl)	P(excl)	P(incl|data)	P(excl|data)	BF_{excl}
Hemifield	0.886	0.114	0.334	0.666	15.537
Eccentricity	0.886	0.114	1.000	6.076e−5	4.733e−4
Sensory Context	0.886	0.114	1.000	7.188e−10	5.599e−9
ageGroup	0.886	0.114	0.996	0.004	0.033
Hemifield ✻ Eccentricity	0.503	0.497	0.065	0.935	14.463
Hemifield ✻ Sensory Context	0.503	0.497	0.049	0.951	19.784
Eccentricity ✻ Sensory Context	0.503	0.497	0.948	0.052	0.055
Hemifield ✻ Eccentricity ✻ Sensory Context	0.120	0.880	0.003	0.997	40.231
Hemifield ✻ ageGroup	0.503	0.497	0.078	0.922	12.043
Eccentricity ✻ ageGroup	0.503	0.497	0.613	0.387	0.640
Hemifield ✻ Eccentricity ✻ ageGroup	0.120	0.880	0.002	0.998	54.690
Sensory Context ✻ ageGroup	0.503	0.497	0.316	0.684	2.193
Hemifield ✻ Sensory Context ✻ ageGroup	0.120	0.880	5.643e−4	0.999	240.977
Eccentricity ✻ Sensory Context ✻ ageGroup	0.120	0.880	0.071	0.929	1.772
Hemifield ✻ Eccentricity ✻ Sensory Context ✻ ageGroup	0.006	0.994	7.324e−7	1.000	8225.462

fMRI results

Decoding spatial representations from fMRI activation patterns along audiovisual pathways

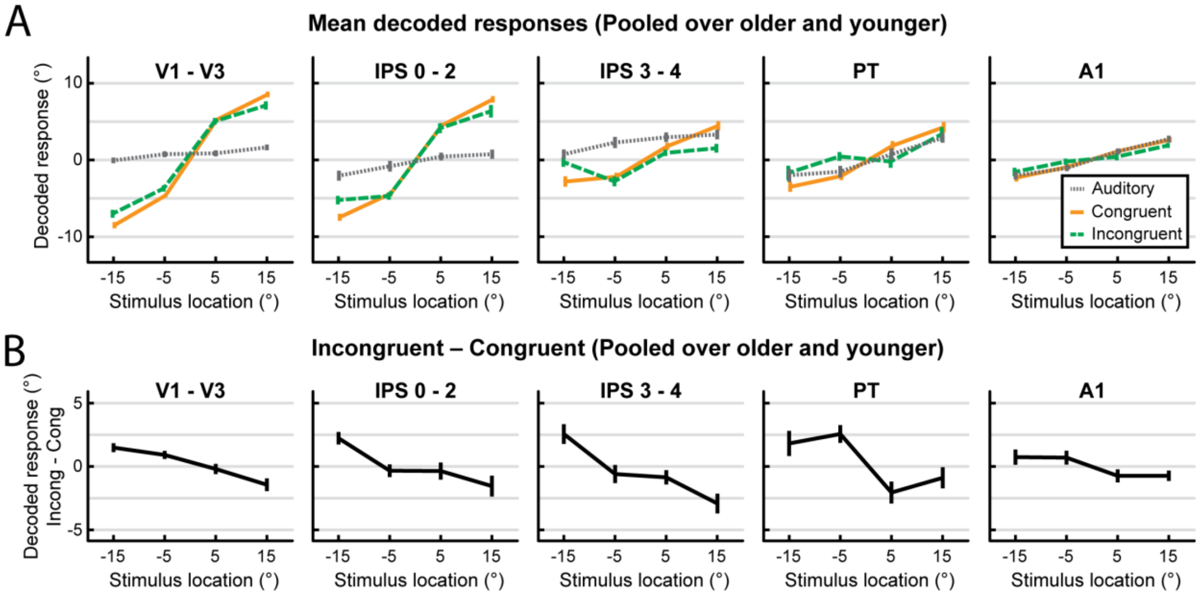

Fig B. fMRI multivariate decoding results (support vector regression) pooled over age groups.
(A) Across-participants mean (±1 SEM) decoded spatial locations for unisensory auditory (grey), audiovisual congruent (orange), and audiovisual incongruent (green) stimuli. (B) Difference between decoded stimulus locations for audiovisual incongruent relative to audiovisual congruent stimuli. Results for five ROIs are shown: visual regions (V1 - V3); posterior intraparietal sulcus (IPS 0 - 2); anterior intraparietal sulcus (IPS 3 - 4); planum temporale (PT); and primary auditory cortex (A1). The data underlying this Figure can be found in S1 Data.

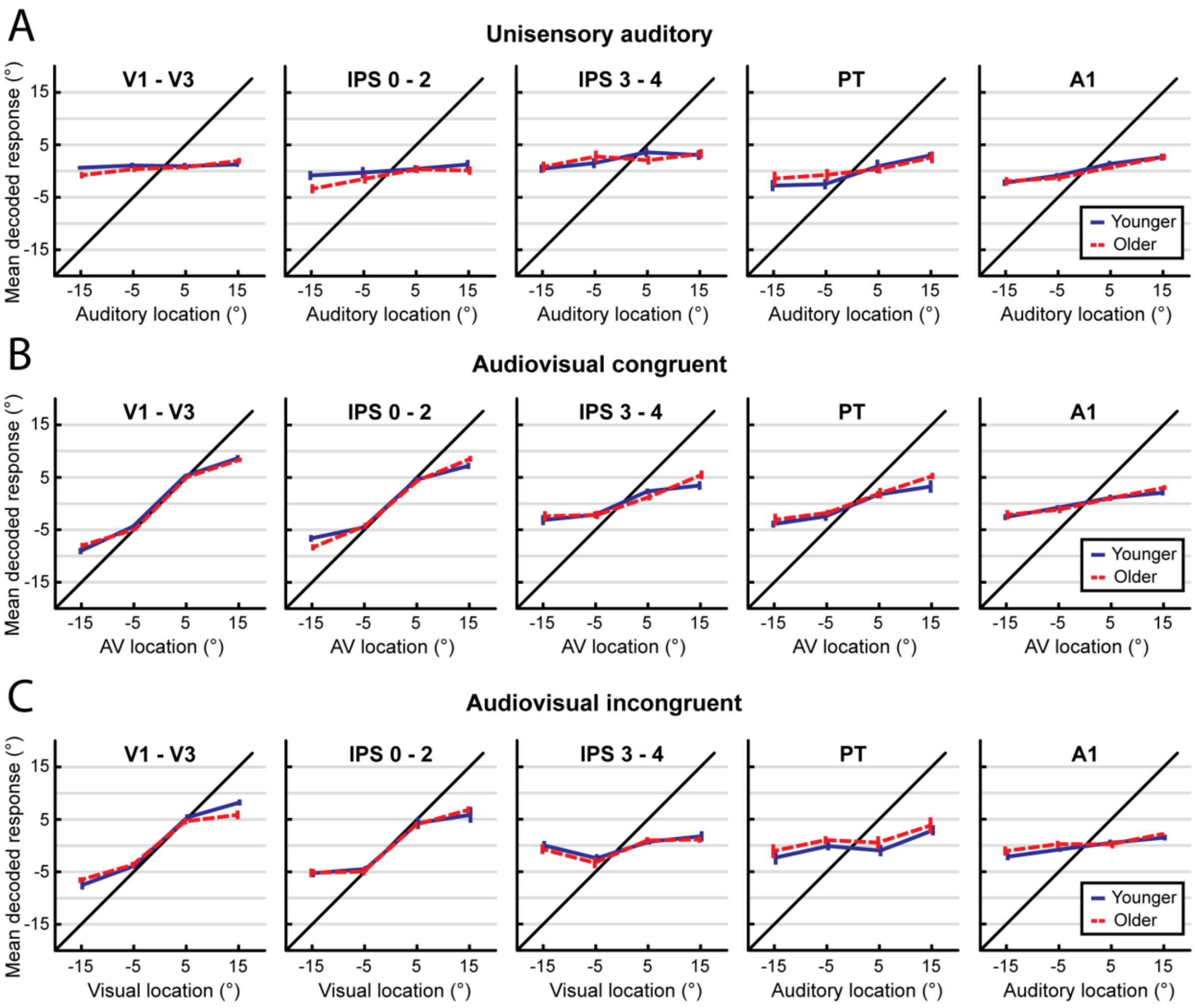

Fig C. fMRI multivariate decoding results (support vector regression) – incongruent stimuli plotted by dominant sensory modality.

Across-participants mean (±1 SEM) decoded spatial locations for younger (blue) and older (red) participants for (A) unisensory auditory, (B) congruent audiovisual, and (C) incongruent audiovisual stimuli. Results for five ROIs are shown: visual regions (V1-V3); posterior intraparietal sulcus (IPS 0-2); anterior intraparietal sulcus (IPS 3-4); planum temporale (PT); and primary auditory cortex (A1). Note that for incongruent conditions, results for all ROIs are plotted according to the location of the stimulus in each region's dominant sensory modality (visual for V1-V3, IPS 0-2, IPS 3-4; auditory for PT and A1). The data underlying this Figure can be found in S1 Data.

Table E. Results of ANOVAs on support vector regression decoded responses in five ROIs (uncorrected* p *values).

	df					
	effect	error	*F*	*p*	η^2_p	BF_{excl}
V1-V3						
**Hemifield**	**1**	**30**	**5.820**	**.022**	**.162**	**0.425**
Hemifield x Age	1	30	0.744	.395	.024	2.458
**Eccentricity**	**1**	**30**	**117.363**	**< .001**	**.796**	**< 0.001**
Eccentricity x Age	1	30	0.874	.357	.028	1.912
**Sensory context**	**1.568**	**47.036**	**328.707**	**< .001**	**.916**	**< 0.001**
**Sensory context x Age**	**1.568**	**47.036**	**5.281**	**.014**	**.150**	**0.355**
Hemifield x Eccentricity	1	30	2.448	.128	.075	1.393
Hemifield x Eccentricity x Age	1	30	0.109	.743	.004	8.696
**Hemifield x Sensory context**	**1.753**	**52.597**	**3.500**	**.043**	**.104**	**0.639**
Hemifield x Sensory context x Age	1.753	52.597	0.594	.534	.019	3.786
**Eccentricity x Sensory context**	**1.620**	**48.588**	**22.205**	**< .001**	**.425**	**< 0.001**
Eccentricity x Sensory context x Age	1.620	48.588	3.165	.061	.095	0.616
Hemifield x Eccentricity x Sensory context	1.666	49.985	1.437	.247	.046	1.645
Hemifield x Eccentricity x Sensory context x Age	1.666	49.985	1.395	.256	.044	37.023
Age	1	30	0.386	.539	.013	1.555
Posterior intraparietal sulcus (IPS 0-2)						
Hemifield	1.000	30.000	.039	.845	.001	35.532
Hemifield x Age	1.000	30.000	.432	.516	.014	53.582
**Eccentricity**	**1.000**	**30.000**	**47.714**	**< .001**	**.614**	**< 0.001**
Eccentricity x Age	1.000	30.000	2.075	.160	.065	6.011
**Sensory context**	**1.656**	**49.671**	**108.823**	**< .001**	**.784**	**< 0.001**
Sensory context x Age	1.656	49.671	.170	.804	.006	13.756
Hemifield x Eccentricity	1.000	30.000	.536	.470	.018	5.447
Hemifield x Eccentricity x Age	1.000	30.000	.170	.683	.006	42.344
Hemifield x Sensory context	1.710	51.315	1.234	.295	.040	5.291
Hemifield x Sensory context x Age	1.710	51.315	1.457	.242	.046	33.293
**Eccentricity x Sensory context**	**1.603**	**48.084**	**9.836**	**< .001**	**.247**	**0.008**
Eccentricity x Sensory context x Age	1.603	48.084	1.140	.318	.037	11.146
**Hemifield x Eccentricity x Sensory context**	**1.934**	**58.032**	**4.956**	**.011**	**.142**	**1.925**
**Hemifield x Eccentricity x Sensory context x Age**	**1.934**	**58.032**	**3.392**	**.042**	**.102**	**> 1000**
Age	1	30	1.845	.185	.058	11.370
Anterior intraparietal sulcus (IPS 3-4)						
**Hemifield**	**1**	**30**	**6.420**	**.017**	**.176**	**< 0.001**
Hemifield x Age	1	30	< .001	.989	< .001	11.163
**Eccentricity**	**1**	**30**	**5.152**	**.031**	**.147**	**0.006**
Eccentricity x Age	1	30	1.894	.179	.059	10.467
**Sensory context**	**1.711**	**51.345**	**14.072**	**< .001**	**.319**	**< 0.001**
Sensory context x Age	1.711	51.345	0.954	.380	.031	18.119

**Hemifield x Eccentricity**	**1**	**30**	**7.857**	**.009**	**.208**	**0.012**
Hemifield x Eccentricity x Age	1	30	1.995	.168	.062	12.914
**Hemifield x Sensory context**	**1.758**	**52.737**	**13.737**	**< .001**	**.314**	**< 0.001**
Hemifield x Sensory context x Age	1.758	52.737	0.437	.623	.014	32.517
**Eccentricity x Sensory context**	**1.841**	**55.234**	**8.495**	**< .001**	**.221**	**0.002**
Eccentricity x Sensory context x Age	1.841	55.234	1.210	.303	.039	34.829
**Hemifield x Eccentricity x Sensory context**	**1.947**	**58.422**	**4.627**	**.014**	**.134**	**0.004**
Hemifield x Eccentricity x Sensory context x Age	1.947	58.422	1.228	.300	.039	92.673
Age	1	30	0.125	.726	.004	18.601
Planum temporale (PT)						
Hemifield	1	30	0.189	.667	.006	9.322
Hemifield x Age	1	30	1.240	.274	.040	14.982
**Eccentricity**	**1**	**30**	**31.000**	**< .001**	**.508**	**0.003**
Eccentricity x Age	1	30	0.112	.740	.004	15.161
**Sensory context**	**1.841**	**55.227**	**10.694**	**< .001**	**.263**	**0.081**
Sensory context x Age	1.841	55.227	1.275	.286	.041	18.890
**Hemifield x Eccentricity**	**1**	**30**	**4.591**	**.040**	**.133**	**3.641**
Hemifield x Eccentricity x Age	1	30	0.077	.783	.003	83.677
Hemifield x Sensory context	1.955	58.650	0.701	.497	.023	14.915
Hemifield x Sensory context x Age	1.955	58.650	0.238	.784	.008	547.346
Eccentricity x Sensory context	1.848	55.427	2.129	.132	.066	2.993
Eccentricity x Sensory context x Age	1.848	55.427	0.285	.735	.009	378.394
Hemifield x Eccentricity x Sensory context	1.971	59.138	0.069	.931	.002	176.626
Hemifield x Eccentricity x Sensory context x Age	1.971	59.138	0.284	.751	.009	> 1000
Age	1	30	0.216	.645	.007	16.997
A1						
Hemifield	1	30	0.173	.680	.006	20.998
Hemifield x Age	1	30	0.334	.568	.011	37.346
**Eccentricity**	**1**	**30**	**21.772**	**< .001**	**.421**	**0.016**
Eccentricity x Age	1	30	0.092	.764	.003	18.084
**Sensory context**	**1.857**	**55.713**	**4.239**	**.022**	**.124**	**4.259**
Sensory context x Age	1.857	55.713	0.646	.517	.021	41.044
Hemifield x Eccentricity	1	30	0.526	.474	.017	18.068
Hemifield x Eccentricity x Age	1	30	3.391	.075	.102	179.325
Hemifield x Sensory context	1.858	55.750	0.009	.989	< .001	72.881
Hemifield x Sensory context x Age	1.858	55.750	1.193	.308	.038	> 1000
Eccentricity x Sensory context	1.995	59.855	0.044	.957	.001	21.128
Eccentricity x Sensory context x Age	1.995	59.855	0.155	.856	.005	> 1000
Hemifield x Eccentricity x Sensory context	1.832	54.949	0.173	.823	.006	> 1000
Hemifield x Eccentricity x Sensory context x Age	1.832	54.949	0.066	.923	.002	> 1000
Age	1	30	0.110	.743	.004	22.037

Greenhouse-Geisser correction applied to all within-subjects tests with $df_{effect} > 1$*. BF_{excl} is based on an equivalent Bayesian ANOVA; greater values indicate more evidence that a given term does not have predictive value within the model (see Materials and Methods for more details).*

Table F. Results of ANOVA on support vector regression decoded responses in ROI V1-V3 – excluding unisensory condition (uncorrected* p *values).

Within Subjects Effects

Cases	Sphericity Correction	Sum of Squares	df	Mean Square	F	p	η^2_p
Hemifield	None	13.166	1.000	13.166	2.348	0.136	0.073
Hemifield ✻ ageGroup	None	3.650	1.000	3.650	0.651	0.426	0.021
Residuals	None	168.215	30.000	5.607			
Eccentricity	None	627.049	1.000	627.049	88.921	1.763e−10	0.748
Eccentricity ✻ ageGroup	None	16.596	1.000	16.596	2.353	0.135	0.073
Residuals	None	211.552	30.000	7.052			
Sensory Context	None	65.184	1.000	65.184	23.857	3.229e−5	0.443
Sensory Context ✻ ageGroup	None	10.073	1.000	10.073	3.687	0.064	0.109
Residuals	None	81.968	30.000	2.732			
Hemifield ✻ Eccentricity	None	13.064	1.000	13.064	4.427	0.044	0.129
Hemifield ✻ Eccentricity ✻ ageGroup	None	0.441	1.000	0.441	0.150	0.702	0.005
Residuals	None	88.523	30.000	2.951			
Hemifield ✻ Sensory Context	None	2.354	1.000	2.354	0.947	0.338	0.031
Hemifield ✻ Sensory Context ✻ ageGroup	None	4.149	1.000	4.149	1.669	0.206	0.053
Residuals	None	74.578	30.000	2.486			
Eccentricity ✻ Sensory Context	None	13.202	1.000	13.202	7.154	0.012	0.193
Eccentricity ✻ Sensory Context ✻ ageGroup	None	0.555	1.000	0.555	0.301	0.587	0.010
Residuals	None	55.364	30.000	1.845			
Hemifield ✻ Eccentricity ✻ Sensory Context	None	1.769	1.000	1.769	0.642	0.429	0.021
Hemifield ✻ Eccentricity ✻ Sensory Context ✻ ageGroup	None	5.600	1.000	5.600	2.031	0.164	0.063
Residuals	None	82.694	30.000	2.756			

Note. Sphericity corrections not available for factors with 2 levels.
Note. Type III Sum of Squares

Between Subjects Effects

Cases	Sum of Squares	df	Mean Square	F	p	η^2_p
ageGroup	27.181	1	27.181	1.927	0.175	0.060
Residuals	423.253	30	14.108			

Note. Type III Sum of Squares

Analysis of Effects

Effects	P(incl)	P(excl)	P(incl|data)	P(excl|data)	BF_{excl}
Hemifield	0.886	0.114	0.747	0.253	2.645
Eccentricity	0.886	0.114	1.000	1.991e−9	1.551e−8
Sensory Context	0.886	0.114	0.999	7.714e−4	0.006
ageGroup	0.886	0.114	0.777	0.223	2.232
Hemifield ✻ Eccentricity	0.503	0.497	0.454	0.546	1.217
Hemifield ✻ Sensory Context	0.503	0.497	0.223	0.777	3.531
Eccentricity ✻ Sensory Context	0.503	0.497	0.689	0.311	0.456
Hemifield ✻ Eccentricity ✻ Sensory Context	0.120	0.880	0.030	0.970	4.457
Hemifield ✻ ageGroup	0.503	0.497	0.191	0.809	4.280
Eccentricity ✻ ageGroup	0.503	0.497	0.384	0.616	1.624
Hemifield ✻ Eccentricity ✻ ageGroup	0.120	0.880	0.017	0.983	7.770
Sensory Context ✻ ageGroup	0.503	0.497	0.385	0.615	1.616
Hemifield ✻ Sensory Context ✻ ageGroup	0.120	0.880	0.014	0.986	9.534
Eccentricity ✻ Sensory Context ✻ ageGroup	0.120	0.880	0.037	0.963	3.563
Hemifield ✻ Eccentricity ✻ Sensory Context ✻ ageGroup	0.006	0.994	8.864e−5	1.000	67.952

Table G. Results of ANOVA on support vector regression decoded responses in ROI IPS0-2 – excluding unisensory condition (uncorrected* p *values).

Within Subjects Effects

Cases	Sphericity Correction	Sum of Squares	df	Mean Square	F	p	η^2_p
Hemifield	None	4.348	1.000	4.348	0.223	0.640	0.007
Hemifield ∗ ageGroup	None	0.007	1.000	0.007	3.766e−4	0.985	1.255e−5
Residuals	None	585.167	30.000	19.506			
Eccentricity	None	342.184	1.000	342.184	66.462	4.228e−9	0.689
Eccentricity ∗ ageGroup	None	17.849	1.000	17.849	3.467	0.072	0.104
Residuals	None	154.457	30.000	5.149			
Sensory Context	None	57.888	1.000	57.888	5.407	0.027	0.153
Sensory Context ∗ ageGroup	None	2.281	1.000	2.281	0.213	0.648	0.007
Residuals	None	321.168	30.000	10.706			
Hemifield ∗ Eccentricity	None	16.029	1.000	16.029	3.334	0.078	0.100
Hemifield ∗ Eccentricity ∗ ageGroup	None	1.578	1.000	1.578	0.328	0.571	0.011
Residuals	None	144.245	30.000	4.808			
Hemifield ∗ Sensory Context	None	2.106e−4	1.000	2.106e−4	3.112e−5	0.996	1.037e−6
Hemifield ∗ Sensory Context ∗ ageGroup	None	0.130	1.000	0.130	0.019	0.891	6.377e−4
Residuals	None	203.054	30.000	6.768			
Eccentricity ∗ Sensory Context	None	56.186	1.000	56.186	8.860	0.006	0.228
Eccentricity ∗ Sensory Context ∗ ageGroup	None	7.888	1.000	7.888	1.244	0.274	0.040
Residuals	None	190.256	30.000	6.342			
Hemifield ∗ Eccentricity ∗ Sensory Context	None	7.558	1.000	7.558	3.352	0.077	0.101
Hemifield ∗ Eccentricity ∗ Sensory Context ∗ ageGroup	None	5.813	1.000	5.813	2.579	0.119	0.079
Residuals	None	67.635	30.000	2.255			

Note. Sphericity corrections not available for factors with 2 levels.
Note. Type III Sum of Squares

Between Subjects Effects

Cases	Sum of Squares	df	Mean Square	F	p	η^2_p
ageGroup	13.450	1	13.450	0.862	0.361	0.028
Residuals	468.173	30	15.606			

Note. Type III Sum of Squares

Analysis of Effects

Effects	P(incl)	P(excl)	P(incl|data)	P(excl|data)	BF_{excl}
Hemifield	0.886	0.114	0.600	0.400	5.191
Eccentricity	0.886	0.114	1.000	4.683e−8	3.648e−7
Sensory Context	0.886	0.114	0.973	0.027	0.218
ageGroup	0.886	0.114	0.667	0.333	3.892
Hemifield ∗ Eccentricity	0.503	0.497	0.268	0.732	2.766
Hemifield ∗ Sensory Context	0.503	0.497	0.161	0.839	5.259
Eccentricity ∗ Sensory Context	0.503	0.497	0.852	0.148	0.175
Hemifield ∗ Eccentricity ∗ Sensory Context	0.120	0.880	0.044	0.956	2.944
Hemifield ∗ ageGroup	0.503	0.497	0.172	0.828	4.869
Eccentricity ∗ ageGroup	0.503	0.497	0.398	0.602	1.531
Hemifield ∗ Eccentricity ∗ ageGroup	0.120	0.880	0.004	0.996	34.462
Sensory Context ∗ ageGroup	0.503	0.497	0.239	0.761	3.228
Hemifield ∗ Sensory Context ∗ ageGroup	0.120	0.880	0.003	0.997	40.187
Eccentricity ∗ Sensory Context ∗ ageGroup	0.120	0.880	0.121	0.879	0.984
Hemifield ∗ Eccentricity ∗ Sensory Context ∗ ageGroup	0.006	0.994	2.446e−5	1.000	246.278

Table H. Results of ANOVA on support vector regression decoded responses in ROI IPS3-4 – excluding unisensory condition (uncorrected* p *values).

Within Subjects Effects

Cases	Sphericity Correction	Sum of Squares	df	Mean Square	F	p	η^2_p
Hemifield	None	0.357	1.000	0.357	0.020	0.889	6.562e−4
Hemifield ∗ ageGroup	None	0.066	1.000	0.066	0.004	0.952	1.212e−4
Residuals	None	544.365	30.000	18.145			
Eccentricity	None	7.608	1.000	7.608	1.314	0.261	0.042
Eccentricity ∗ ageGroup	None	1.611	1.000	1.611	0.278	0.602	0.009
Residuals	None	173.698	30.000	5.790			
Sensory Context	None	131.247	1.000	131.247	21.997	5.582e−5	0.423
Sensory Context ∗ ageGroup	None	0.846	1.000	0.846	0.142	0.709	0.005
Residuals	None	179.000	30.000	5.967			
Hemifield ∗ Eccentricity	None	107.423	1.000	107.423	11.694	0.002	0.280
Hemifield ∗ Eccentricity ∗ ageGroup	None	10.365	1.000	10.365	1.128	0.297	0.036
Residuals	None	275.595	30.000	9.187			
Hemifield ∗ Sensory Context	None	13.597	1.000	13.597	1.357	0.253	0.043
Hemifield ∗ Sensory Context ∗ ageGroup	None	12.912	1.000	12.912	1.289	0.265	0.041
Residuals	None	300.617	30.000	10.021			
Eccentricity ∗ Sensory Context	None	108.587	1.000	108.587	12.422	0.001	0.293
Eccentricity ∗ Sensory Context ∗ ageGroup	None	10.962	1.000	10.962	1.254	0.272	0.040
Residuals	None	262.239	30.000	8.741			
Hemifield ∗ Eccentricity ∗ Sensory Context	None	4.768	1.000	4.768	0.659	0.423	0.021
Hemifield ∗ Eccentricity ∗ Sensory Context ∗ ageGroup	None	20.434	1.000	20.434	2.824	0.103	0.086
Residuals	None	217.039	30.000	7.235			

Note. Sphericity corrections not available for factors with 2 levels.
Note. Type III Sum of Squares

Between Subjects Effects

Cases	Sum of Squares	df	Mean Square	F	p	η^2_p
ageGroup	2.114	1	2.114	0.117	0.734	0.004
Residuals	539.838	30	17.995			

Note. Type III Sum of Squares

Analysis of Effects

Effects	P(incl)	P(excl)	P(incl|data)	P(excl|data)	BF_{excl}
Hemifield	0.886	0.114	0.895	0.105	0.917
Eccentricity	0.886	0.114	0.982	0.018	0.139
Sensory Context	0.886	0.114	1.000	3.445e−4	0.003
ageGroup	0.886	0.114	0.396	0.604	11.882
Hemifield ∗ Eccentricity	0.503	0.497	0.852	0.148	0.176
Hemifield ∗ Sensory Context	0.503	0.497	0.351	0.649	1.872
Eccentricity ∗ Sensory Context	0.503	0.497	0.947	0.053	0.057
Hemifield ∗ Eccentricity ∗ Sensory Context	0.120	0.880	0.101	0.899	1.212
Hemifield ∗ ageGroup	0.503	0.497	0.101	0.899	9.041
Eccentricity ∗ ageGroup	0.503	0.497	0.096	0.904	9.482
Hemifield ∗ Eccentricity ∗ ageGroup	0.120	0.880	0.010	0.990	13.535
Sensory Context ∗ ageGroup	0.503	0.497	0.093	0.907	9.827
Hemifield ∗ Sensory Context ∗ ageGroup	0.120	0.880	0.005	0.995	26.902
Eccentricity ∗ Sensory Context ∗ ageGroup	0.120	0.880	0.009	0.991	14.413
Hemifield ∗ Eccentricity ∗ Sensory Context ∗ ageGroup	0.006	0.994	1.107e−4	1.000	54.403

Table I. Results of ANOVA on support vector regression decoded responses in ROI PT – excluding unisensory condition (uncorrected* p *values).

Within Subjects Effects

Cases	Sphericity Correction	Sum of Squares	df	Mean Square	F	p	η^2_p
Hemifield	None	22.779	1.000	22.779	0.564	0.458	0.018
Hemifield * ageGroup	None	73.472	1.000	73.472	1.819	0.187	0.057
Residuals	None	1211.521	30.000	40.384			
Eccentricity	None	361.678	1.000	361.678	36.481	1.247e−6	0.549
Eccentricity * ageGroup	None	0.775	1.000	0.775	0.078	0.782	0.003
Residuals	None	297.428	30.000	9.914			
Sensory Context	None	213.534	1.000	213.534	23.168	3.946e−5	0.436
Sensory Context * ageGroup	None	0.460	1.000	0.460	0.050	0.825	0.002
Residuals	None	276.509	30.000	9.217			
Hemifield * Eccentricity	None	23.163	1.000	23.163	2.472	0.126	0.076
Hemifield * Eccentricity * ageGroup	None	1.881	1.000	1.881	0.201	0.657	0.007
Residuals	None	281.110	30.000	9.370			
Hemifield * Sensory Context	None	7.672	1.000	7.672	0.491	0.489	0.016
Hemifield * Sensory Context * ageGroup	None	0.996	1.000	0.996	0.064	0.802	0.002
Residuals	None	468.311	30.000	15.610			
Eccentricity * Sensory Context	None	14.452	1.000	14.452	1.332	0.258	0.043
Eccentricity * Sensory Context * ageGroup	None	5.175	1.000	5.175	0.477	0.495	0.016
Residuals	None	325.496	30.000	10.850			
Hemifield * Eccentricity * Sensory Context	None	0.685	1.000	0.685	0.060	0.808	0.002
Hemifield * Eccentricity * Sensory Context * ageGroup	None	6.064	1.000	6.064	0.529	0.472	0.017
Residuals	None	343.603	30.000	11.453			

Note. Sphericity corrections not available for factors with 2 levels.
Note. Type III Sum of Squares

Between Subjects Effects

Cases	Sum of Squares	df	Mean Square	F	p	η^2_p
ageGroup	0.657	1	0.657	0.023	0.880	7.764e−4
Residuals	846.135	30	28.204			

Note. Type III Sum of Squares

Analysis of Effects

Effects	P(incl)	P(excl)	P(incl|data)	P(excl|data)	BF_{excl}
Hemifield	0.886	0.114	0.510	0.490	7.496
Eccentricity	0.886	0.114	1.000	8.729e−5	6.800e−4
Sensory Context	0.886	0.114	0.997	0.003	0.021
ageGroup	0.886	0.114	0.366	0.634	13.475
Hemifield * Eccentricity	0.503	0.497	0.199	0.801	4.079
Hemifield * Sensory Context	0.503	0.497	0.134	0.866	6.525
Eccentricity * Sensory Context	0.503	0.497	0.299	0.701	2.378
Hemifield * Eccentricity * Sensory Context	0.120	0.880	0.004	0.996	38.186
Hemifield * ageGroup	0.503	0.497	0.098	0.902	9.278
Eccentricity * ageGroup	0.503	0.497	0.079	0.921	11.720
Hemifield * Eccentricity * ageGroup	0.120	0.880	0.003	0.997	42.204
Sensory Context * ageGroup	0.503	0.497	0.080	0.920	11.604
Hemifield * Sensory Context * ageGroup	0.120	0.880	0.002	0.998	68.892
Eccentricity * Sensory Context * ageGroup	0.120	0.880	0.002	0.998	73.267
Hemifield * Eccentricity * Sensory Context * ageGroup	0.006	0.994	6.830e−7	1.000	8819.420

Table J. Results of ANOVA on support vector regression decoded responses in ROI A1 – excluding unisensory condition (uncorrected* p *values).

Within Subjects Effects

Cases	Sphericity Correction	Sum of Squares	df	Mean Square	F	p	η^2_p
Hemifield	None	1.429	1.000	1.429	0.163	0.689	0.005
Hemifield ✻ ageGroup	None	12.174	1.000	12.174	1.389	0.248	0.044
Residuals	None	262.895	30.000	8.763			
Eccentricity	None	127.828	1.000	127.828	17.913	2.009e−4	0.374
Eccentricity ✻ ageGroup	None	1.204	1.000	1.204	0.169	0.684	0.006
Residuals	None	214.080	30.000	7.136			
Sensory Context	None	33.792	1.000	33.792	5.346	0.028	0.151
Sensory Context ✻ ageGroup	None	6.676	1.000	6.676	1.056	0.312	0.034
Residuals	None	189.627	30.000	6.321			
Hemifield ✻ Eccentricity	None	0.158	1.000	0.158	0.031	0.861	0.001
Hemifield ✻ Eccentricity ✻ ageGroup	None	8.135	1.000	8.135	1.615	0.214	0.051
Residuals	None	151.080	30.000	5.036			
Hemifield ✻ Sensory Context	None	0.003	1.000	0.003	6.775e−4	0.979	2.258e−5
Hemifield ✻ Sensory Context ✻ ageGroup	None	1.735	1.000	1.735	0.401	0.531	0.013
Residuals	None	129.782	30.000	4.326			
Eccentricity ✻ Sensory Context	None	0.005	1.000	0.005	0.001	0.969	4.982e−5
Eccentricity ✻ Sensory Context ✻ ageGroup	None	0.546	1.000	0.546	0.181	0.674	0.006
Residuals	None	90.442	30.000	3.015			
Hemifield ✻ Eccentricity ✻ Sensory Context	None	0.009	1.000	0.009	0.002	0.963	7.129e−5
Hemifield ✻ Eccentricity ✻ Sensory Context ✻ ageGroup	None	0.575	1.000	0.575	0.144	0.707	0.005
Residuals	None	119.842	30.000	3.995			

Note. Sphericity corrections not available for factors with 2 levels.
Note. Type III Sum of Squares

Between Subjects Effects

Cases	Sum of Squares	df	Mean Square	F	p	η^2_p
ageGroup	0.744	1	0.744	0.093	0.763	0.003
Residuals	240.472	30	8.016			

Note. Type III Sum of Squares

Analysis of Effects

Effects	P(incl)	P(excl)	P(incl|data)	P(excl|data)	BF_{excl}
Hemifield	0.886	0.114	0.332	0.668	15.676
Eccentricity	0.886	0.114	0.995	0.005	0.037
Sensory Context	0.886	0.114	0.741	0.259	2.716
ageGroup	0.886	0.114	0.341	0.659	15.029
Hemifield ✻ Eccentricity	0.503	0.497	0.070	0.930	13.483
Hemifield ✻ Sensory Context	0.503	0.497	0.052	0.948	18.411
Eccentricity ✻ Sensory Context	0.503	0.497	0.145	0.855	5.985
Hemifield ✻ Eccentricity ✻ Sensory Context	0.120	0.880	4.996e−4	1.000	272.207
Hemifield ✻ ageGroup	0.503	0.497	0.051	0.949	18.978
Eccentricity ✻ ageGroup	0.503	0.497	0.084	0.916	11.095
Hemifield ✻ Eccentricity ✻ ageGroup	0.120	0.880	0.002	0.998	81.749
Sensory Context ✻ ageGroup	0.503	0.497	0.086	0.914	10.767
Hemifield ✻ Sensory Context ✻ ageGroup	0.120	0.880	8.392e−4	0.999	161.984
Eccentricity ✻ Sensory Context ✻ ageGroup	0.120	0.880	0.001	0.999	106.705
Hemifield ✻ Eccentricity ✻ Sensory Context ✻ ageGroup	0.006	0.994	2.687e−7	1.000	22417.911

Table K. Results of ANOVA on support vector regression decoded responses, all conditions labelled by the location of the auditory stimulus, in ROI V1-V3 (uncorrected* p *values).

Within Subjects Effects

Cases	Sphericity Correction	Sum of Squares	df	Mean Square	F	p	η^2_p
Hemifield	None	63.958	1.000	63.958	5.820	0.022	0.162
Hemifield ∗ ageGroup	None	8.175	1.000	8.175	0.744	0.395	0.024
Residuals	None	329.672	30.000	10.989			
Eccentricity	None	30.606	1.000	30.606	16.530	3.187e−4	0.355
Eccentricity ∗ ageGroup	None	3.798	1.000	3.798	2.051	0.162	0.064
Residuals	None	55.548	30.000	1.852			
Sensory Context	None	9876.695[a]	2.000[a]	4938.348[a]	583.725[a]	4.725e−40[a]	0.951
	Greenhouse-Geisser	9876.695	1.329	7430.983	583.725	2.002e−27	0.951
Sensory Context ∗ ageGroup	None	28.608[a]	2.000[a]	14.304[a]	1.691[a]	0.193[a]	0.053
	Greenhouse-Geisser	28.608	1.329	21.524	1.691	0.202	0.053
Residuals	None	507.603	60.000	8.460			
	Greenhouse-Geisser	507.603	39.874	12.730			
Hemifield ∗ Eccentricity	None	9.008	1.000	9.008	2.448	0.128	0.075
Hemifield ∗ Eccentricity ∗ ageGroup	None	0.402	1.000	0.402	0.109	0.743	0.004
Residuals	None	110.410	30.000	3.680			
Hemifield ∗ Sensory Context	None	27.607	2.000	13.803	3.500	0.037	0.104
	Greenhouse-Geisser	27.607	1.753	15.746	3.500	0.043	0.104
Hemifield ∗ Sensory Context ∗ ageGroup	None	4.689	2.000	2.344	0.594	0.555	0.019
	Greenhouse-Geisser	4.689	1.753	2.674	0.594	0.534	0.019
Residuals	None	236.631	60.000	3.944			
	Greenhouse-Geisser	236.631	52.597	4.499			
Eccentricity ∗ Sensory Context	None	629.392[a]	2.000[a]	314.696[a]	73.734[a]	6.855e−17[a]	0.711
	Greenhouse-Geisser	629.392	1.401	449.342	73.734	1.622e−12	0.711
Eccentricity ∗ Sensory Context ∗ ageGroup	None	18.742[a]	2.000[a]	9.371[a]	2.196[a]	0.120[a]	0.068
	Greenhouse-Geisser	18.742	1.401	13.381	2.196	0.138	0.068
Residuals	None	256.080	60.000	4.268			
	Greenhouse-Geisser	256.080	42.021	6.094			
Hemifield ∗ Eccentricity ∗ Sensory Context	None	5.833[a]	2.000[a]	2.916[a]	1.437[a]	0.246[a]	0.046
	Greenhouse-Geisser	5.833	1.666	3.501	1.437	0.247	0.046
Hemifield ∗ Eccentricity ∗ Sensory Context ∗ ageGroup	None	5.664[a]	2.000[a]	2.832[a]	1.395[a]	0.256[a]	0.044
	Greenhouse-Geisser	5.664	1.666	3.399	1.395	0.256	0.044
Residuals	None	121.806	60.000	2.030			
	Greenhouse-Geisser	121.806	49.985	2.437			

Note. Sphericity corrections not available for factors with 2 levels.
Note. Type III Sum of Squares
[a] Mauchly's test of sphericity indicates that the assumption of sphericity is violated (p < .05).

Between Subjects Effects

Cases	Sum of Squares	df	Mean Square	F	p	η^2_p
ageGroup	22.388	1	22.388	6.421	0.017	0.176
Residuals	104.594	30	3.486			

Note. Type III Sum of Squares

Analysis of Effects

Effects	P(incl)	P(excl)	P(incl|data)	P(excl|data)	BF_{excl}
Hemifield	0.886	0.114	0.962	0.038	0.307
Eccentricity	0.886	0.114	1.000	0.000	0.000
Sensory Context	0.886	0.114	1.000	0.000	0.000
ageGroup	0.886	0.114	0.891	0.109	0.953
Hemifield ∗ Eccentricity	0.503	0.497	0.307	0.693	2.289
Hemifield ∗ Sensory Context	0.503	0.497	0.743	0.257	0.350
Eccentricity ∗ Sensory Context	0.503	0.497	1.000	6.439e−15	6.517e−15
Hemifield ∗ Eccentricity ∗ Sensory Context	0.120	0.880	0.064	0.936	1.979
Hemifield ∗ ageGroup	0.503	0.497	0.198	0.802	4.102
Eccentricity ∗ ageGroup	0.503	0.497	0.216	0.784	3.664
Hemifield ∗ Eccentricity ∗ ageGroup	0.120	0.880	0.010	0.990	14.163
Sensory Context ∗ ageGroup	0.503	0.497	0.262	0.738	2.847
Hemifield ∗ Sensory Context ∗ ageGroup	0.120	0.880	0.014	0.986	9.388
Eccentricity ∗ Sensory Context ∗ ageGroup	0.120	0.880	0.047	0.953	2.746
Hemifield ∗ Eccentricity ∗ Sensory Context ∗ ageGroup	0.006	0.994	6.025e−5	1.000	99.985

Table L. Results of ANOVA on support vector regression decoded responses, all conditions labelled by the location of the auditory stimulus, in ROI IPS0-2 (uncorrected* p *values).

Within Subjects Effects

Cases	Sphericity Correction	Sum of Squares	df	Mean Square	F	p	η^2_p
Hemifield	None	1.404	1.000	1.404	0.039	0.845	0.001
Hemifield ∗ ageGroup	None	15.597	1.000	15.597	0.432	0.516	0.014
Residuals	None	1083.488	30.000	36.116			
Eccentricity	None	74.615	1.000	74.615	9.093	0.005	0.233
Eccentricity ∗ ageGroup	None	6.343	1.000	6.343	0.773	0.386	0.025
Residuals	None	246.162	30.000	8.205			
Sensory Context	None	7983.382[a]	2.000[a]	3991.691[a]	357.210[a]	4.734e−34[a]	0.923
	Greenhouse-Geisser	7983.382	1.472	5424.696	357.210	1.085e−25	0.923
Sensory Context ∗ ageGroup	None	17.490[a]	2.000[a]	8.745[a]	0.783[a]	0.462[a]	0.025
	Greenhouse-Geisser	17.490	1.472	11.884	0.783	0.427	0.025
Residuals	None	670.477	60.000	11.175			
	Greenhouse-Geisser	670.477	44.150	15.186			
Hemifield ∗ Eccentricity	None	3.119	1.000	3.119	0.536	0.470	0.018
Hemifield ∗ Eccentricity ∗ ageGroup	None	0.989	1.000	0.989	0.170	0.683	0.006
Residuals	None	174.652	30.000	5.822			
Hemifield ∗ Sensory Context	None	27.954	2.000	13.977	1.234	0.298	0.040
	Greenhouse-Geisser	27.954	1.710	16.343	1.234	0.295	0.040
Hemifield ∗ Sensory Context ∗ ageGroup	None	33.003	2.000	16.502	1.457	0.241	0.046
	Greenhouse-Geisser	33.003	1.710	19.295	1.457	0.242	0.046
Residuals	None	679.692	60.000	11.328			
	Greenhouse-Geisser	679.692	51.315	13.246			
Eccentricity ∗ Sensory Context	None	342.773	2.000	171.386	37.388	2.859e−11	0.555
	Greenhouse-Geisser	342.773	1.719	199.423	37.388	5.376e−10	0.555
Eccentricity ∗ Sensory Context ∗ ageGroup	None	19.547	2.000	9.773	2.132	0.127	0.066
	Greenhouse-Geisser	19.547	1.719	11.372	2.132	0.135	0.066
Residuals	None	275.042	60.000	4.584			
	Greenhouse-Geisser	275.042	51.565	5.334			
Hemifield ∗ Eccentricity ∗ Sensory Context	None	27.243	2.000	13.622	4.956	0.010	0.142
	Greenhouse-Geisser	27.243	1.934	14.084	4.956	0.011	0.142
Hemifield ∗ Eccentricity ∗ Sensory Context ∗ ageGroup	None	18.644	2.000	9.322	3.392	0.040	0.102
	Greenhouse-Geisser	18.644	1.934	9.638	3.392	0.042	0.102
Residuals	None	164.902	60.000	2.748			
	Greenhouse-Geisser	164.902	58.032	2.842			

Note. Sphericity corrections not available for factors with 2 levels.
Note. Type III Sum of Squares
[a] Mauchly's test of sphericity indicates that the assumption of sphericity is violated ($p < .05$).

Between Subjects Effects

Cases	Sum of Squares	df	Mean Square	F	p	η^2_p
ageGroup	10.700	1	10.700	1.296	0.264	0.041
Residuals	247.692	30	8.256			

Note. Type III Sum of Squares

Analysis of Effects

Effects	P(incl)	P(excl)	P(incl|data)	P(excl|data)	BF_{excl}
Hemifield	0.886	0.114	0.035	0.965	214.875
Eccentricity	0.886	0.114	1.000	5.236e−10	4.079e−9
Sensory Context	0.886	0.114	1.000	6.328e−15	4.929e−14
ageGroup	0.886	0.114	0.365	0.635	13.541
Hemifield ∗ Eccentricity	0.503	0.497	0.019	0.981	51.097
Hemifield ∗ Sensory Context	0.503	0.497	0.014	0.986	71.679
Eccentricity ∗ Sensory Context	0.503	0.497	1.000	4.165e−9	4.215e−9
Hemifield ∗ Eccentricity ∗ Sensory Context	0.120	0.880	0.008	0.992	16.911
Hemifield ∗ ageGroup	0.503	0.497	0.002	0.998	441.362
Eccentricity ∗ ageGroup	0.503	0.497	0.072	0.928	13.112
Hemifield ∗ Eccentricity ∗ ageGroup	0.120	0.880	5.475e−5	1.000	2484.759
Sensory Context ∗ ageGroup	0.503	0.497	0.072	0.928	13.031
Hemifield ∗ Sensory Context ∗ ageGroup	0.120	0.880	4.126e−5	1.000	3297.367
Eccentricity ∗ Sensory Context ∗ ageGroup	0.120	0.880	0.009	0.991	14.998
Hemifield ∗ Eccentricity ∗ Sensory Context ∗ ageGroup	0.006	0.994	1.907e−5	1.000	315.879

Table M. Results of ANOVA on support vector regression decoded responses, all conditions labelled by the location of the auditory stimulus, in ROI IPS3-4 (uncorrected* p *values).

Within Subjects Effects

Cases	Sphericity Correction	Sum of Squares	df	Mean Square	F	p	η^2_p
Hemifield	None	222.567	1.000	222.567	6.420	0.017	0.176
Hemifield ✻ ageGroup	None	0.007	1.000	0.007	1.882e−4	0.989	6.272e−6
Residuals	None	1040.003	30.000	34.667			
Eccentricity	None	133.932	1.000	133.932	14.910	5.582e−4	0.332
Eccentricity ✻ ageGroup	None	23.922	1.000	23.922	2.663	0.113	0.082
Residuals	None	269.489	30.000	8.983			
Sensory Context	None	1122.659[a]	2.000[a]	561.329[a]	46.863[a]	5.523e−13[a]	0.610
	Greenhouse-Geisser	1122.659	1.443	777.964	46.863	5.445e−10	0.610
Sensory Context ✻ ageGroup	None	10.099[a]	2.000[a]	5.050[a]	0.422[a]	0.658[a]	0.014
	Greenhouse-Geisser	10.099	1.443	6.998	0.422	0.593	0.014
Residuals	None	718.692	60.000	11.978			
	Greenhouse-Geisser	718.692	43.292	16.601			
Hemifield ✻ Eccentricity	None	42.995	1.000	42.995	7.857	0.009	0.208
Hemifield ✻ Eccentricity ✻ ageGroup	None	10.916	1.000	10.916	1.995	0.168	0.062
Residuals	None	164.174	30.000	5.472			
Hemifield ✻ Sensory Context	None	416.108	2.000	208.054	13.737	1.225e−5	0.314
	Greenhouse-Geisser	416.108	1.758	236.707	13.737	3.395e−5	0.314
Hemifield ✻ Sensory Context ✻ ageGroup	None	13.224	2.000	6.612	0.437	0.648	0.014
	Greenhouse-Geisser	13.224	1.758	7.523	0.437	0.623	0.014
Residuals	None	908.722	60.000	15.145			
	Greenhouse-Geisser	908.722	52.737	17.231			
Eccentricity ✻ Sensory Context	None	10.438	2.000	5.219	1.026	0.365	0.033
	Greenhouse-Geisser	10.438	1.927	5.418	1.026	0.362	0.033
Eccentricity ✻ Sensory Context ✻ ageGroup	None	3.009	2.000	1.504	0.296	0.745	0.010
	Greenhouse-Geisser	3.009	1.927	1.562	0.296	0.737	0.010
Residuals	None	305.122	60.000	5.085			
	Greenhouse-Geisser	305.122	57.798	5.279			
Hemifield ✻ Eccentricity ✻ Sensory Context	None	80.089	2.000	40.045	4.627	0.014	0.134
	Greenhouse-Geisser	80.089	1.947	41.126	4.627	0.014	0.134
Hemifield ✻ Eccentricity ✻ Sensory Context ✻ ageGroup	None	21.251	2.000	10.625	1.228	0.300	0.039
	Greenhouse-Geisser	21.251	1.947	10.912	1.228	0.300	0.039
Residuals	None	519.306	60.000	8.655			
	Greenhouse-Geisser	519.306	58.422	8.889			

Note. Sphericity corrections not available for factors with 2 levels.
Note. Type III Sum of Squares
[a] Mauchly's test of sphericity indicates that the assumption of sphericity is violated (p < .05).

Between Subjects Effects

Cases	Sum of Squares	df	Mean Square	F	p	η^2_p
ageGroup	9.763	1	9.763	1.470	0.235	0.047
Residuals	199.275	30	6.642			

Note. Type III Sum of Squares

Analysis of Effects

Effects	P(incl)	P(excl)	P(incl|data)	P(excl|data)	BF_{excl}
Hemifield	0.886	0.114	1.000	6.272e−6	4.886e−5
Eccentricity	0.886	0.114	0.997	0.003	0.020
Sensory Context	0.886	0.114	1.000	2.887e−15	2.248e−14
ageGroup	0.886	0.114	0.375	0.625	12.997
Hemifield ✻ Eccentricity	0.503	0.497	0.940	0.060	0.064
Hemifield ✻ Sensory Context	0.503	0.497	1.000	7.407e−5	7.497e−5
Eccentricity ✻ Sensory Context	0.503	0.497	0.838	0.162	0.196
Hemifield ✻ Eccentricity ✻ Sensory Context	0.120	0.880	0.820	0.180	0.030
Hemifield ✻ ageGroup	0.503	0.497	0.116	0.884	7.721
Eccentricity ✻ ageGroup	0.503	0.497	0.137	0.863	6.373
Hemifield ✻ Eccentricity ✻ ageGroup	0.120	0.880	0.015	0.985	8.824
Sensory Context ✻ ageGroup	0.503	0.497	0.055	0.945	17.290
Hemifield ✻ Sensory Context ✻ ageGroup	0.120	0.880	0.004	0.996	32.773
Eccentricity ✻ Sensory Context ✻ ageGroup	0.120	0.880	0.003	0.997	49.925
Hemifield ✻ Eccentricity ✻ Sensory Context ✻ ageGroup	0.006	0.994	5.996e−5	1.000	100.465

Identification of neural systems involved in spatial localisation of audiovisual signals

Effects of stimuli and task relative to fixation

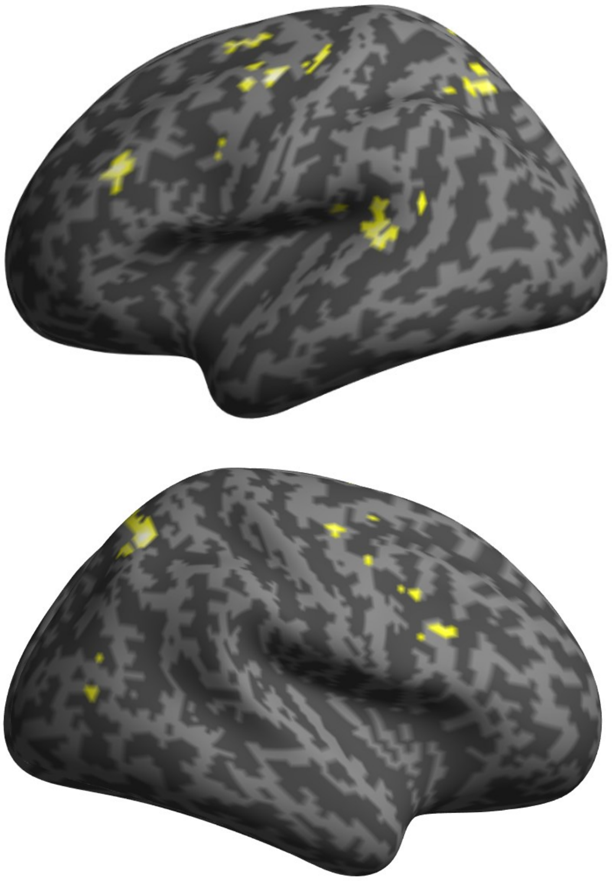

Fig D. Age-related activation increases in response to congruent stimuli.
Activation increases for older relative to younger adults, for congruent stimuli only, are rendered on an inflated canonical brain. Height threshold p < .05, whole-brain familywise-error corrected. Full results can be viewed within the SPM.mat file, also in Supporting Information. The data underlying this Figure can be found in S2 Data.

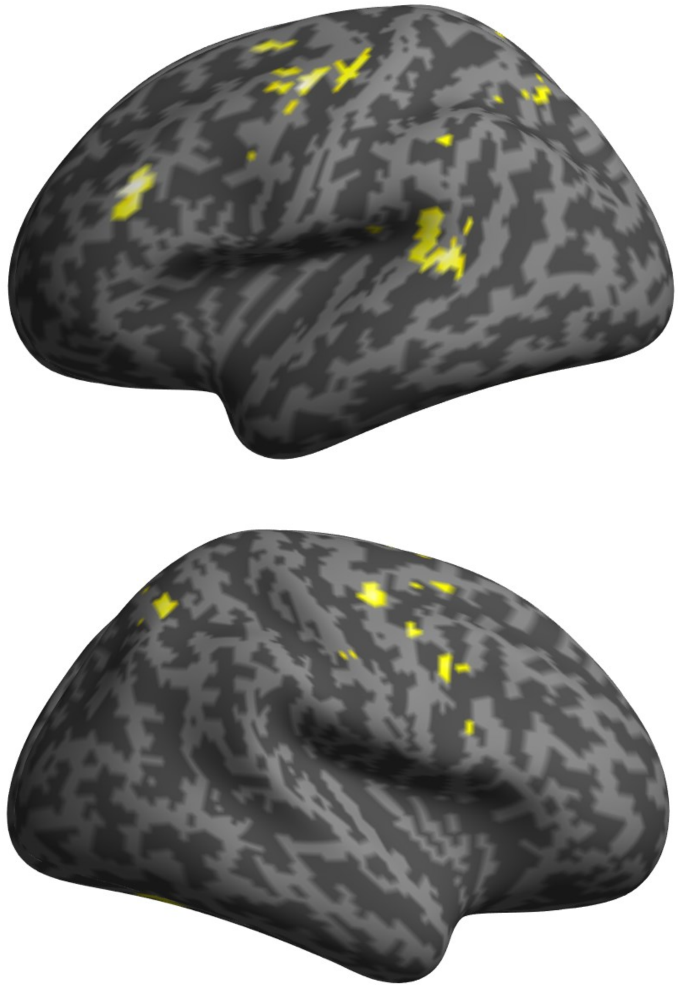

Fig E. Age-related activation increases in response to incongruent stimuli.

Activation increases for older relative to younger adults, for incongruent stimuli only, are rendered on an inflated canonical brain. Height threshold p < .05, whole-brain familywise-error corrected. Full results can be viewed within the SPM.mat file, also in Supporting Information. The data underlying this Figure can be found in S2 Data.

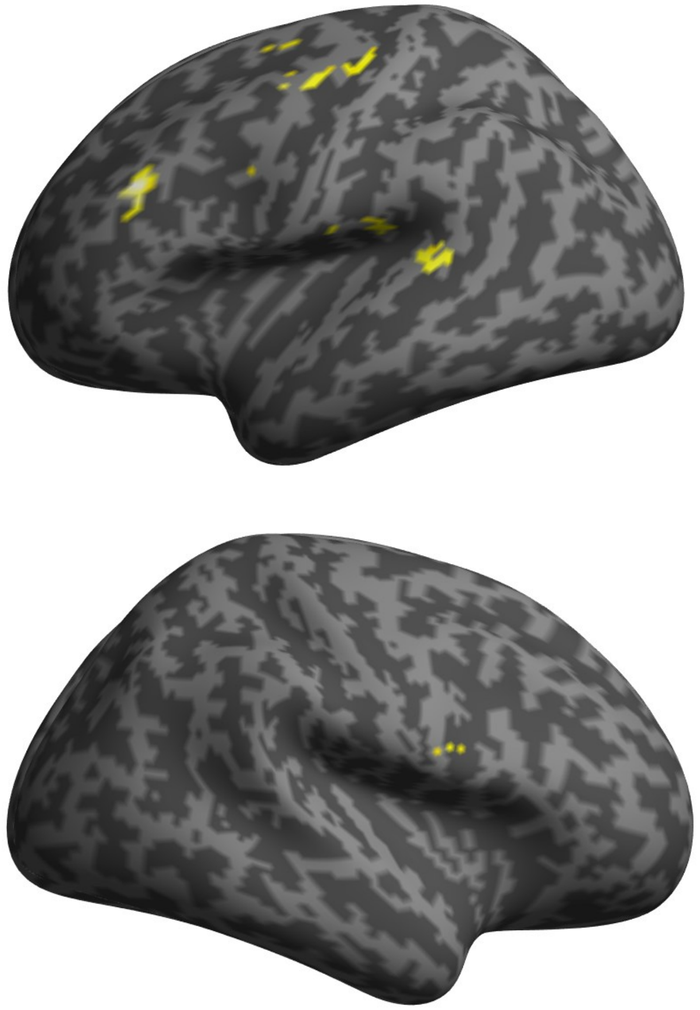

Fig F. Age-related activation increases in response to unisensory auditory stimuli. *Activation increases for older relative to younger adults, for unisensory auditory stimuli only, are rendered on an inflated canonical brain. Height threshold p < .05, whole-brain familywise-error corrected. Full results can be viewed within the SPM.mat file, also in Supporting Information. The data underlying this Figure can be found in S2 Data.*

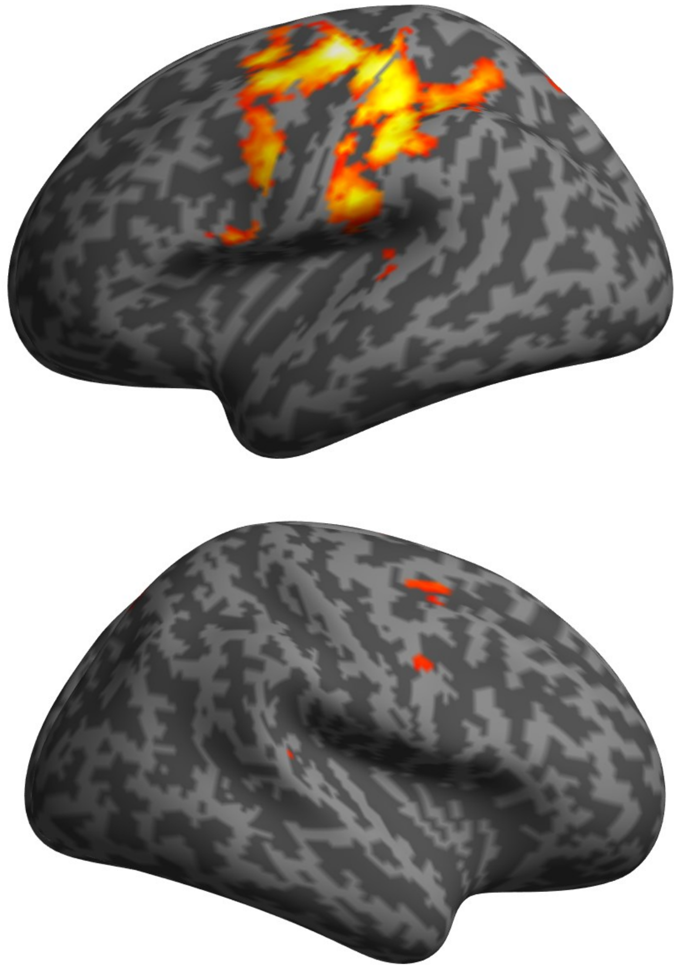

Fig G. Task-related activations in younger adults only.

Task-related activations (i.e., in response to all stimuli, relative to fixation) in the younger group are rendered on an inflated canonical brain. Height threshold p < .05, whole-brain familywise-error corrected. Full results can be viewed within the SPM.mat file, also in Supporting Information. The data underlying this Figure can be found in S2 Data.

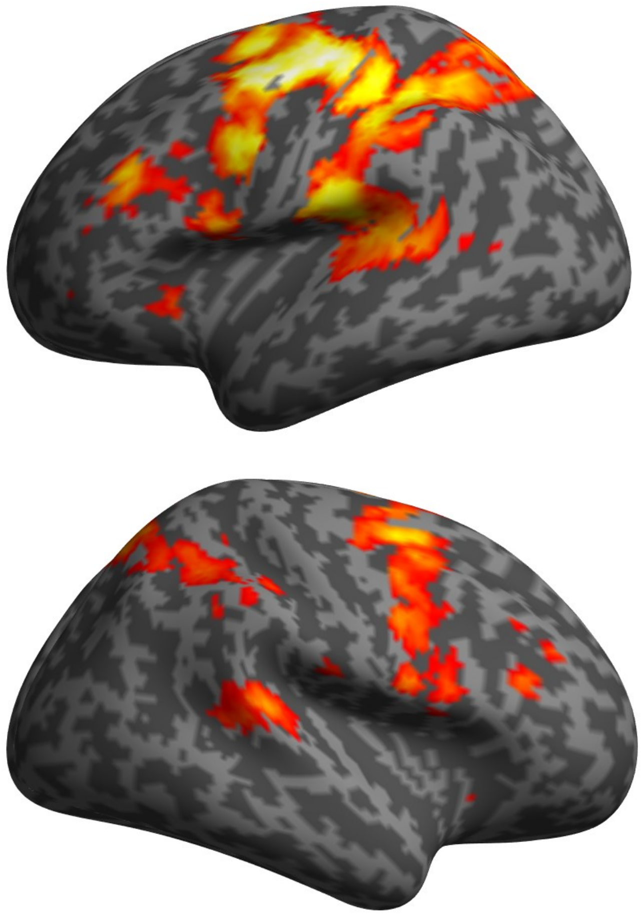

Fig H. Task-related activations in older adults only.

Task-related activations (i.e., in response to all stimuli, relative to fixation) in the older group are rendered on an inflated canonical brain. Height threshold p < .05, whole-brain familywise-error corrected. Full results can be viewed within the SPM.mat file, also in Supporting Information. The data underlying this Figure can be found in S2 Data.

Quantifying stimulus-relevant information in task-related BOLD responses

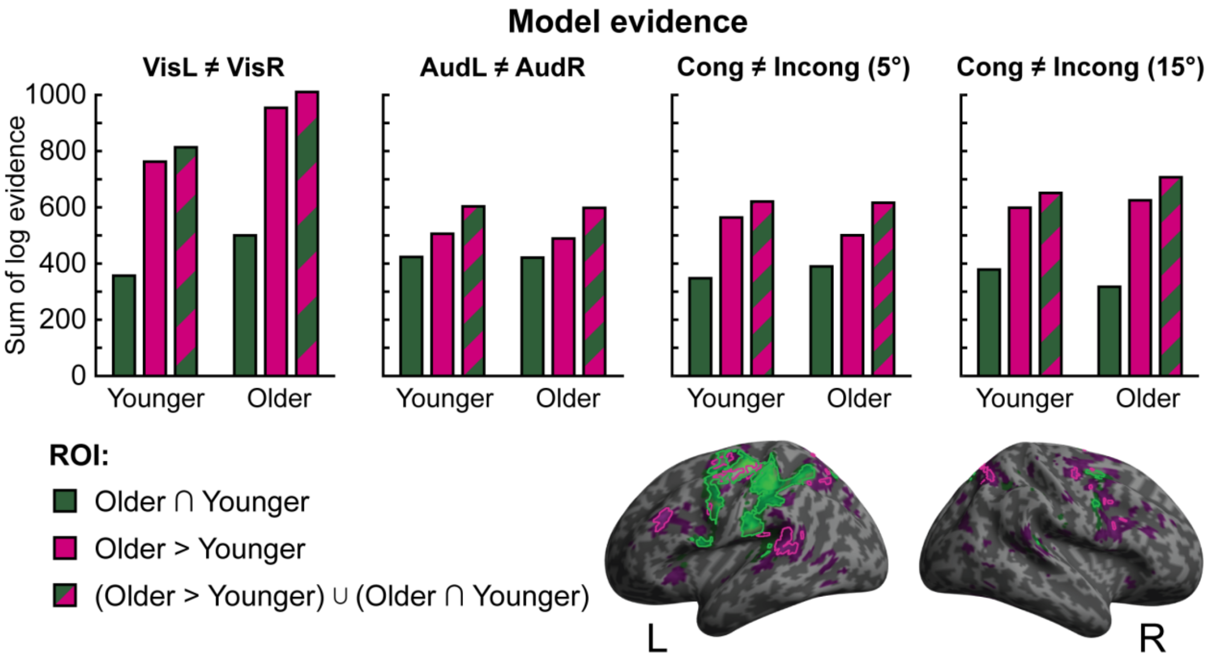

Fig I. Results of multivariate Bayesian decoding analysis (model evidence).
Comparison of three sets of regions ([O∩Y], [O>Y] or union of both: [O>Y] ∪ [O∩Y]) in their ability to predict stimulus-related target variables: visual location, auditory location, congruent/incongruent at 5° and congruent/incongruent at 15°. Log model evidence values, summed across participants, are shown for each target variable and each set of regions. The data underlying this Figure can be found in S1 Data.

Table N. Comparison between age groups in the "boost" in encoded information provided by the combined set of regions ([O>Y] ∪ [O∩Y]) over [O∩Y] alone

Independent Samples T-Test

	W	df	p
diff_VisLR_UnionMINUSOconjY	116.000		0.669
diff_AudLR_UnionMINUSOconjY	126.000		0.956
diff_CongIncong5_UnionMINUSOconjY	139.000		0.696
diff_CongIncong15_UnionMINUSOconjY	69.000		0.026

Note. Mann-Whitney U test.

Bayesian Mann-Whitney U Test

	BF_{01}	W	Rhat
diff_VisLR_UnionMINUSOconjY	2.415	116.000	1.000
diff_AudLR_UnionMINUSOconjY	2.866	126.000	1.000
diff_CongIncong5_UnionMINUSOconjY	2.568	139.000	1.000
diff_CongIncong15_UnionMINUSOconjY	0.616	69.000	1.000

Note. Result based on data augmentation algorithm with 5 chains of 10000 iterations.

Materials and Methods

Screening and training session (outside the scanner)

Participants attended a total of three sessions on three separate days. In the first session, prior to any MRI scanning, they underwent behavioural screening and training. MRI scanning took place in sessions two and three for participants who passed screening.

Auditory spatial classification task

First, a left/right forced-choice spatial classification task was used to assess participants' ability to accurately localise auditory stimuli. Participants were presented on each trial with an auditory stimulus randomly at one of ten locations between -15° and 15° azimuth (-15°, -10°, -5°, -3°, -1°, 1°, 3°, 5°, 10°, 15°) and indicated via a two-choice button press whether they perceived the sound as coming from the left or right. For each observer, we computed the proportion of 'perceived right' for each of the ten locations. Using a Nelder-Mead optimisation algorithm, as implemented in the Palamedes toolbox (Version 1.10.3) [67] for MATLAB, we fitted a four-parameter (α, β, λ, γ) cumulative Gaussian function to these data. The parameters of this function were the mean of the distribution α (i.e., point of subjective equality, PSE), the slope parameter β, (i.e., the reciprocal of the participant's spatial uncertainty), and the lapse parameters λ and γ (i.e., the probability of incorrectly responding right when stimuli were perceived to be on the left, and vice versa). We calculated each participant's just-noticeable difference, a measure of spatial uncertainty, as the reciprocal of the fitted slope (JND = $1/\beta$).

The JND, PSE, and absolute PSE (PSE_{abs}; magnitude of left/right bias, disregarding direction) were entered into separate independent-samples t-tests to compare older and younger adults. We observed no significant differences in spatial precision or left/right bias between age groups; only a non-significant trend of larger JNDs (lower auditory spatial

reliability) was evident in older adults: JND $t(30) = 1.532$, $p = .136$, $d = 0.542$, $BF_{01} = 1.228$; PSE $t(30) = 0.527$, $p = .602$, $d = 0.186$, $BF_{01} = 2.673$; PSE_{abs} $t(30) = 0.244$, $p = .809$, $d = .086$, $BF_{01} = 2.907$. This suggests approximately comparable localisation performance for older and younger participant groups in an unspeeded auditory spatial classification task. Note that a JND of less than 10° was also specified as an inclusion criterion; all participants met this requirement.

Auditory localisation training

Second, participants were trained to learn the mapping between the auditory locations (-15°, -5°, 5°, and 15°) and the four corresponding buttons used in the main ventriloquist paradigm. Via a four-choice key press, participants localised a sound that was presented randomly from one of the four locations on each trial. Feedback was provided after each response: correct responses were rewarded with a green square presented at the correct/responded location; incorrect responses resulted in a red square presented at the responded location, followed by a green square presented at the correct location. Participants completed up to five 20-trial blocks, stopping early if localisation accuracy (i.e., correct button responses) reached 90% in any block.

Spatial localisation training and eye tracking

Third, participants completed two blocks of the spatial ventriloquist paradigm used during the two MRI scanning sessions. During these blocks, the scanner noise recorded from an fMRI sequence was played over speakers at a level that approximately matched that experienced in the scanner (after adjustment for headphone attenuation). Analyses of data from this session are included in the Supporting Information.

Because many older adults find remaining still for extended periods of time challenging and painful, we were unable to perform reliable eye tracking during fMRI

scanning due to the associated extra setup and calibration times. Therefore, to minimise the possibility of eye movement confounds in the fMRI data, we instead screened participants beforehand for their ability to maintain central fixation during the task. Throughout the two blocks of the ventriloquist paradigm, participants' eye movements were recorded via a Tobii EyeX eye tracker. A custom MATLAB script was used to remove blinks and identify saccades. For each participant, the peak location (i.e., furthest from fixation) of every recorded saccade was entered as the outcome variable in a linear regression analysis, with visual stimulus location as the predictor variable. Any participant for whom the stimulus location significantly predicted peak saccade location was not invited back for the MRI sessions. In this way, participants with stimulus-driven saccades were excluded from the study (seven older adults, three younger; see Participants subsection).
